# Identification of lactylation and its hub genes in contributing immune activation and renal allograft fibrosis by integrative bioinformatics and machine learning

**DOI:** 10.3389/fimmu.2026.1741864

**Published:** 2026-02-10

**Authors:** Feifei Yuan, Jiewu Huang, Dantong Huang, Kexin Li, Shan Zhou, Lili Zhou

**Affiliations:** 1 Division of Nephrology, Nanfang Hospital, Southern Medical University, Guangzhou, China; 2 National Clinical Research Center for Kidney Disease, Guangzhou, China; 3 State Key Laboratory of Multi-organ Injury Prevention and Treatment, Guangzhou, China; 4 Guangdong Provincial Institute of Nephrology, Guangzhou, China; 5 Guangdong Provincial Key Laboratory of Renal Failure Research, Guangzhou, China; 6 Central Laboratory, The Fifth Affiliated Hospital, Southern Medical University, Guangzhou, Guangdong, China

**Keywords:** bioinformatics, immune infiltration, kidney transplant, lactylation, renal allograft fibrosis

## Abstract

**Background:**

Late graft loss due to chronic renal allograft fibrosis remains a major challenge after kidney transplantation. Excessive immune-cell activation is a key driver of allograft fibrosis; however, the underlying mechanisms remain incompletely understood. Recent studies have implicated lactylation, a post-translational protein modification derived from lactate, in immune activation. Nonetheless, the role of lactylation in renal allograft fibrosis has not been systematically explored.

**Methods:**

Transcriptomic datasets from kidney transplant recipients with and without interstitial fibrosis/tubular atrophy (IFTA) were obtained from the GEO database. Differentially expressed genes were intersected with lactylation-related genes (LRGs) to identify differentially expressed LRGs (DELRGs). Functional enrichment analyses were performed to explore associated biological processes and pathways. Weighted gene co-expression network analysis (WGCNA) combined with multiple machine-learning algorithms was used to screen for hub genes. A lactylation-related risk score (LRS) was constructed and validated across independent cohorts, and its predictive performance was evaluated by receiver operating characteristic (ROC) analysis. Single-nucleus RNA sequencing (snRNA-seq) data from allograft biopsies (GSE195718) were processed with Seurat and Harmony for clustering and annotation; cell type–specific hub LRG expression and lactylation scores were profiled. Two murine renal fibrosis models were established to validate the expression of hub genes and to assess their associations with immune-cell infiltration.

**Results:**

We identified five hub LRGs—*IKZF1, PDLIM1, S100A11, STAT4* and *SLC2A3*—that were strongly associated with renal allograft fibrosis. These genes were closely linked to pathways related to lactate metabolism, immune activation and oxidative stress. The LRS based on these genes showed robust predictive accuracy in both the training and validation cohorts. In addition, snRNA-seq of allograft biopsies localized hub LRGs predominantly to immune-lineage and stromal clusters with higher lactylation scores in IFTA samples; concordantly, immune-infiltration analyses revealed significant positive correlations between hub LRGs and multiple immune-cell subsets. Furthermore, these hub genes were upregulated in murine models of renal fibrosis.

**Conclusion:**

This study identified five lactylation-related hub genes that are closely associated with immune-cell infiltration and exhibit strong predictive performance, suggesting their potential as diagnostic biomarkers and therapeutic targets in renal allograft fibrosis.

## 1 Introduction

Kidney transplantation is the preferred renal replacement therapy for patients with end-stage kidney disease (1, 2). Although graft survival rates are improved than before, with 1-year and 10-year rates exceeding 90% and 50%, respectively, long-term graft survival is not satisfactory yet because of chronic allograft injury (CAI) (3–5). CAI is driven by immune rejection, ischemia–reperfusion injury, and metabolic abnormalities, etc., resulting in interstitial fibrosis and tubular atrophy (IFTA) (6). However, the underlying mechanisms of CAI have not been fully clarified.

Epigenetic modifications have recently been implicated in post-transplant immune activation, particularly DNA methylation and histone acetylation, and, more recently, lysine lactylation (7–10). Unlike classical acetylation and methylation, which broadly encode intracellular acetyl-CoA and S-adenosylmethionine availability, lactylation is directly derived from lactate and therefore acts as a metabolite-specific epigenetic “code” for glycolytic flux and lactate accumulation (10–12). Notably, many pro-inflammatory and activated immune cells that accumulate in the transplanted kidney—such as effector T cells, classically activated (M1) macrophages and mature dendritic cells—are highly glycolytic (13). In these glycolysis-addicted populations, lactylation shows temporal dynamics distinct from acetylation: acetylation changes occur early, whereas lactylation preferentially accumulates in the late phase of sustained glycolysis and high lactate, functioning as a “lactate clock” that links chronic metabolic stress to gene expression (2, 12). In the setting of chronic allograft injury, characterized by long-lasting low-grade inflammation and progressive fibrosis in a lactate-rich microenvironment, this temporal and metabolic specificity makes lactylation a particularly relevant epigenetic layer beyond traditional acetylation and methylation. However, the roles of lactylation-related genes (LRGs) in renal allograft fibrosis remain poorly understood.

Metabolic imbalance is frequently occurred after transplantation (14). As a result, metabolic imbalance further exacerbates the loss of protein homeostasis. For example, post-transplant diabetes mellitus (PTDM) causes glomerular and tubular injury, but also triggers microvascular rarefaction and tissue hypoxia, thereby driving a metabolic shift from fatty acid β-oxidation (FAO) to glycolysis (14, 15). Of note, highly elevated lactate promotes protein lactylation on histone or non-histone proteins to change the characteristics of cells. Reports showed histone lactylation, such as H3K18la and H4K12la, activated NF-κB and RhoA/ROCK signaling, the key pro-inflammatory and pro-fibrotic pathways (16, 17). Non-histone substrate lactylation, such as Fis1-K20la and ACSF2-K182la, also disrupts mitochondrial dynamics and cellular homeostasis to accelerate fibrotic progression (18, 19). These findings suggest protein lactylation is possibly a key mechanism for kidney allograft fibrosis, as an epigenetic mediator (20). Hence, the authentic role of lactylation and the LRGs should be widely explored by a comprehensive bioinformatics analysis.

Although kidney biopsy remains the gold standard for assessing renal allograft pathology and predicting graft loss (21), its invasive nature and limited feasibility for repeated sampling restrict its application in long-term monitoring (22). By contrast, the integration of bioinformatics and machine-learning techniques can process large-scale, high-dimensional biological data and help identify potential biomarkers and critical molecular pathways (23). However, the systematic bioinformatics analyses in renal allograft fibrosis are still lacking.

**Figure 1:**
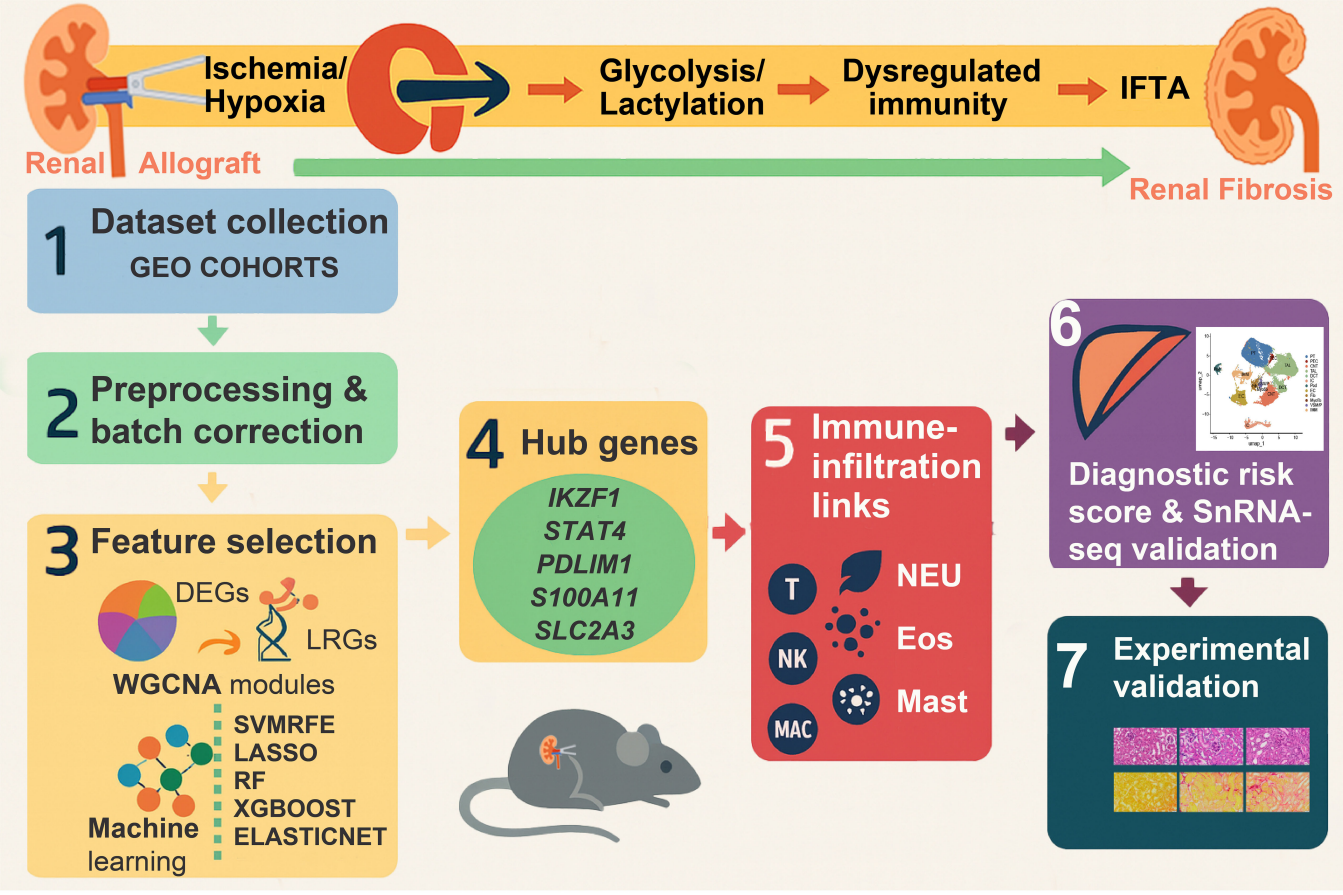
Integrative framework for lactylation-centered biomarkers in renal allograft fibrosis (IFTA). (1) Kidney allograft biopsy transcriptome datasets were collected from GEO. (2) Preprocessing and batch correction were performed. (3) Feature selection combined DEGs, LRGs, WGCNA modules, and ML filters (SVM, LASSO, RF, XGBoost, and Elastic Net). (4) Five hub LRGs were identified—*IKZF1, PDLIM1, S100A11, STAT4*, and SLC2A3. (5) Immune-infiltration associations were mapped for T cells, NK cells, macrophages, neutrophils, eosinophils, and mast cells, among others. (6) A hub-gene diagnostic risk score was constructed and externally validated by ROC/AUC. (7) Findings were experimentally validated in a murine renal-fibrosis model.

In this study, we integrated transcriptomic datasets of renal allograft fibrosis. Through weighted gene co-expression network analysis (WGCNA) and machine learning algorithms, we identified five hub lactylation-related genes (*IKZF1, PDLIM1, S100A11, STAT4*, and *SLC2A3*), and found their diagnosis potential in immune activation. Our findings provide novel insights into the molecular mechanisms of kidney allograft fibrosis and highlight promising biomarkers for prediction. The detailed workflow is depicted in **Figure 1**.

## 2 Materials and methods

### 2.1 Dataset acquisition

Kidney allograft biopsy transcriptomic data were retrieved from the GEO database (https://www.ncbi.nlm.nih.gov/geo/). Three datasets (GSE44131, GSE53605 and GSE76882) were integrated as the training cohort, and GSE72925 was analyzed separately as an external validation cohort. Sample classification into IFTA and non-IFTA groups was based on histopathological diagnosis. Specifically, within each dataset, probe IDs were mapped to official gene symbols using the “gene_assignment” field (GSE44131: GPL6244) or the “Gene Symbol” column (GSE53605: GPL571; GSE76882: GPL13158; GSE72925: GPL570). The resulting gene-level expression matrices were used as provided in GEO, which are background-corrected and quantile-normalized on log_2_ scale according to the original submissions. No additional within-dataset normalization was applied. Clinical information was further obtained from the Nephroseq v5 online database. Detailed dataset characteristics are provided in **Supplementary Table S1**.

Lactylation-related genes (LRGs) were collected from three sources. First, predefined gene sets were retrieved from the Molecular Signatures Database (MSigDB; https://www.gsea-msigdb.org/gsea/msigdb/index.jsp).Second, previously reported regulators of lysine lactylation were included, consisting of seven “writers” and six “erasers” (24–27). Third, based on the study by Wan et al., who developed a mass spectrometry–based diagnostic ion approach to systematically characterize lactylated substrates in human cells (28, 29), we incorporated 327 lactylated proteins into our candidate list. After integration and removal of duplicates, a total of 709 LRGs were obtained and are listed in **Supplementary Table S2**.

Single-nucleus RNA sequencing (snRNA-seq) data were obtained from GEO (GSE195718), comprising biopsies from six patients with IFTA and three patients with stable graft function showing normal or nonspecific histopathology (30).

### 2.2 Data preprocessing and identification of differentially expressed LRGs

For integrative analyses across GSE76882, GSE53605, and GSE44131, we first intersected genes present in all datasets and merged the matrices by gene symbol. Batch effects attributable to dataset of origin were further adjusted using the SVA package “ComBat” with study ID as batch and disease status (Normal vs IFTA) as a covariate (31). Differential expression analysis was conducted on the training datasets with the “limma” package, and results were visualized as volcano plots using “ggplot2”. Genes with *P* < 0.05 and |log2FC| > 0.5 were considered differentially expressed genes (DEGs), a threshold commonly used in microarray-based transcriptomic studies to capture modest but biologically meaningful expression changes while maintaining a reasonable false training rate (32, 33). Subsequently, DEGs were intersected with lactylation-related genes (LRGs) to obtain differentially expressed LRGs (DELRGs) for further analysis.

For snRNA-seq data, raw matrices for the nine samples were imported into Seurat v4.3.0 (Read10X) and merged. Nuclei were retained if they expressed 400–5,000 genes and had<2.5% mitochondrial transcripts. Data were normalized and integrated with Harmony within the Seurat workflow to correct batch effects. Clusters were called on the integrated space, and was used to determine cluster markers conserved across conditions(logFC > 0.25, FDR< 0.05). The top 10 markers per cluster guided manual annotation against a published human kidney atlas (34). The full marker-gene lists together with final cell-type labels are provided in **Supplementary Table S4** and visualized in the bubble plot (see Results section). UMAP was used for dimensionality reduction and visualization. For cell-level lactylation signature scoring, the LRGs set above was applied with Seurat::AddModuleScore on the Harmony-integrated object. Cluster-level summaries were obtained by averaging cell-level scores within each cluster.

### 2.3 Pathway and process enrichment and network analysis

**Figure 2:**
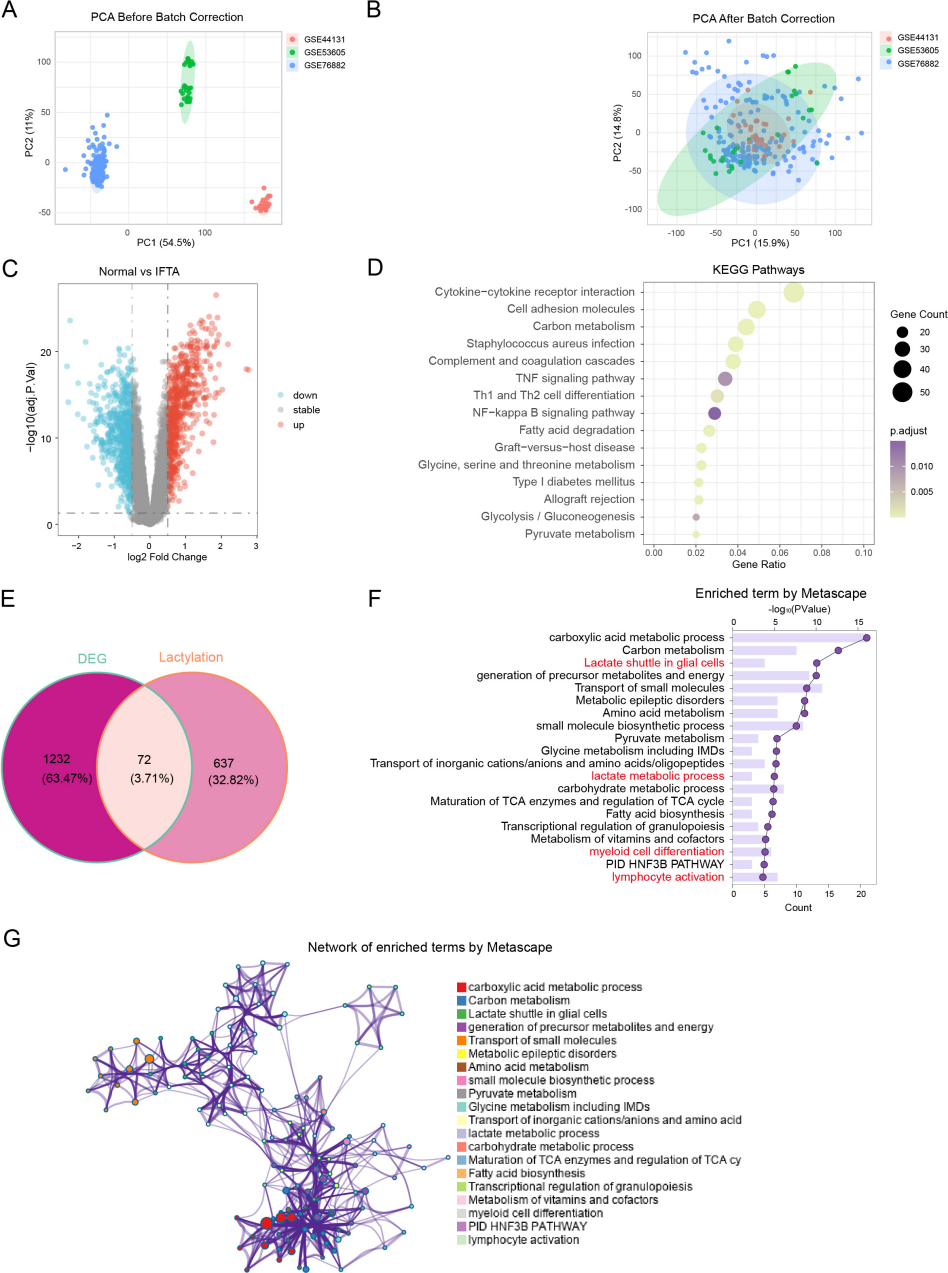
Integrated transcriptomics reveals immune–metabolic dysregulation in renal allograft fibrosis. **(A, B)** PCA plots before and after batch effect correction across three GEO datasets (GSE44131, GSE53605 and GSE76882). **(C)** Volcano plot showing DEGs between IFTA and Normal samples (red, upregulated; blue, downregulated). **(D)** KEGG pathway enrichment of DEGs, highlighting immune- and metabolism-related processes. **(E)** Venn diagram of DEGs and lactylation-related genes (LRGs). **(F)** Bar and line (dual-axis) plot of enriched pathways, indicating enrichment in lactate metabolism and immune pathways. **(G)** Network visualization of enriched pathways, demonstrating crosstalk between lactate metabolism and immune cell activation and differentiation.

Functional enrichment analyses were performed using several complementary approaches. Gene Ontology (GO) (35), Kyoto Encyclopedia of Genes and Genomes (KEGG) (36), and Reactome pathway analyses were implemented with the R package “clusterProfiler”. Gene set enrichment analysis (GSEA) was further applied to investigate underlying biological pathways, with significance defined as adjusted P< 0.05. In parallel, enrichment analysis was also performed using Metascape (http://metascape.org), which integrates multiple ontology sources and generates pathway clusters and functional interaction networks based on gene overlap. The top 20 clusters with their representative enriched terms, as well as the corresponding bar graph and network visualization, are presented in **Figure 2**.

### 2.4 Weighted gene co-expression network analysis

Weighted gene co-expression network analysis was conducted using the “WGCNA” R package to identify gene modules associated with renal allograft fibrosis (37). Hierarchical clustering was first applied to all samples to detect and remove outliers. An appropriate soft-thresholding power was then selected based on the criterion of scale-free topology (R² > 0.85) to ensure reliable network construction. Gene modules were identified using a dynamic tree-cutting algorithm with a minimum module size of 50. Module eigengenes were subsequently correlated with clinical traits (Normal vs. IFTA) to determine modules most relevant to fibrosis. Finally, genes from the module most significantly associated with IFTA were intersected with DEGs and LRGs to obtain fibrosis-related lactylation candidates for downstream analyses.

To assess the stability and reproducibility of the co-expression modules, we applied the “modulePreservation” function in WGCNA using the merged training network (combined GSE44131, GSE53605 and GSE76882) as the reference and each individual dataset as the test set. For each reference–test pair, 200 permutations were performed to obtain preservation statistics. Module preservation was summarized by the Zsummary statistic and interpreted according to the criteria proposed by Langfelder et al. (Zsummary< 2, no evidence of preservation; 2–10, weak to moderate preservation; >10, strong preservation) (38).

### 2.5 Machine learning–based screening of hub genes

Five machine-learning algorithms were applied to screen lactylation-related genes associated with renal allograft fibrosis in the merged training cohort that combined all eligible samples from GSE76882, GSE53605, and GSE44131. For the random forest (RF) model, gene importance scores were calculated from a 500-tree forest using the mean decrease in Gini index, and the out-of-bag error rate was used as an internal estimate of classification performance. LASSO regression with a binomial link was performed using ten-fold cross-validation to select optimal predictors by minimizing cross-validated classification error and binomial deviance. SVM-RFE was implemented with a linear support vector machine and used five-fold cross-validation; features were iteratively eliminated, and the optimal feature subset was determined by the maximum cross-validated classification accuracy and minimum error rate (39). XGBoost gradient-boosting models were trained with 200 boosting rounds (max_depth = 3, eta = 0.1, subsample = 0.9, colsample_bytree = 0.8), and the gain-based feature importance was employed to evaluate the contribution of each feature to model performance. ElasticNet regression combined L1 and L2 regularization and was applied ten-fold cross-validation to obtain stable feature selection. To reduce overfitting and enhance robustness, genes consistently selected across all five algorithms were defined as robust hub genes for further validation and construction of the lactylation-related risk score model.

For each algorithm, we first derived a candidate LRG set (RF and XGBoost: top-ranked genes by importance; LASSO and ElasticNet: genes with non-zero coefficients at the optimal penalty λ; SVM-RFE: features contained in the optimal subset). We then constructed the intersection across these five candidate sets and defined “robust” hub LRGs strictly as genes that were selected concurrently by all five algorithms. Genes present in only a subset of models (e.g., three or four algorithms) were not carried forward to downstream analyses.

### 2.6 Construction of the lactylation-related risk score model

The lactylation-related risk score model was established using LASSO regression (40), which identified five hub genes with the highest predictive value. The risk score was defined as the weighted sum of gene expression levels and their corresponding coefficients:


$$Risk\;Score\;=\sum_{i=1}^{n}\beta_{i}\times Exp_{i}$$

According to this formula, the final model incorporated five hub lactylation-related genes, and the individual score was calculated as:


$$\begin{array}{l} LRS\;=\;(0.6957\times STAT4)\;+\;(0.6615\times PDLIM1)\;+\;(0.8860\times S100A11)\;+\;\\ \left(1.5352\times IKZF1)\;+\;(0.1249\times SLC2A3\right) \end{array}$$

Diagnostic performance of the LRS in both the discovery cohort and the independent validation cohort GSE72925 was evaluated by receiver operating characteristic (ROC) analysis, and area under the curve (AUC), accuracy, sensitivity, and specificity were calculated. These performance metrics are summarized in **Supplementary Table S3**.

### 2.7 Analysis of immune cell infiltration

Immune cell infiltration was evaluated using single-sample Gene Set Enrichment Analysis (ssGSEA), which estimates the enrichment level of immune-related gene signatures within individual samples. Gene sets representing 28 immune cell types were obtained from previously published studies (41). The analysis was implemented with the “GSVA” R package, which transformed the gene expression profiles into immune cell–specific enrichment scores, allowing for comparative assessment of immune infiltration across samples.

### 2.8 Animal models

The murine renal ischemia–reperfusion injury (IRI) model was established to induce renal fibrosis, following previously published protocols (42, 43). Male C57BL/6 mice (8–12 weeks old, 20–25 g) were purchased from the Southern Medical University Animal Center (Guangzhou, China) and maintained under specific pathogen-free (SPF) conditions with free access to food and water. Mice were randomly assigned to three groups (sham, mild IRI, and severe IRI) and anesthetized with sodium pentobarbital (30 mg/kg, i.p.). In the sham group, the renal pedicle was exposed for 20–30 min without clamping. In the mild IRI group, the unilateral renal pedicle was occluded for 20 min, whereas in the severe IRI group, ischemia was induced for 30 min. After reperfusion, animals were monitored and sacrificed 14 days after surgery, and kidney tissues were harvested for downstream analyses. For the unilateral ureteral obstruction (UUO) model, the left ureter was exposed and double-ligated to induce obstruction; sham mice underwent the same procedure without ligation. Mice were sacrificed 7 days after surgery and kidneys were collected.

All animal experiments were performed in accordance with the AVMA Guidelines for the Euthanasia of Animals (2020). Briefly, animals were exposed to CO_2_ using a gradual-fill technique at 30–70% of chamber volume per minute until loss of consciousness, followed by cervical dislocation to ensure death. Personnel performing physical methods were trained and demonstrated proficiency. All animal experiments were performed in accordance with the policies of the Animal Care and Use Committee and approved by the Animal Ethics Committee of the Nanfang Hospital, Southern Medical University (Approval No. IACUC-LAC-20250923-001).

### 2.9 Histology and immunofluorescence

After euthanasia, kidneys were fixed in 4% paraformaldehyde at 4°C (overnight), dehydrated through graded ethanol, cleared in xylene, and embedded in paraffin. Serial sections (3 μm) were cut on a rotary microtome. Routine hematoxylin–Eosin (H&E) staining was performed for general morphology and Sirius Red staining (BASO, BA4356) was applied to visualize interstitial collagen according to the manufacturers’ protocols.

For immunofluorescence, paraffin sections were deparaffinized, rehydrated, and subjected to heat-induced epitope retrieval (citrate buffer, pH 6.0). After blocking with 5% bovine serum albumin, slides were incubated with primary reagents at 4°C overnight: anti-FN (F6140, Sigma), anti-F4/80 (GB11027, Servicebio). Appropriate fluorophore-conjugated secondary antibodies were applied the next day, and nuclei were counterstained with DAPI.

### 2.10 Histology and immunofluorescence quantification

For each group, 5–6 mice were analyzed. For each mouse kidney, 10 randomly selected nonoverlapping high-power fields were quantified. Fibrotic lesions were expressed as the percentage of positive staining area relative to the total tissue area. For immunofluorescence, regions of interest (ROIs) were defined as the kidney cortex, and ROIs were quantified for mean fluorescence intensity (MFI) or positive cell counts using ImageJ. All quantifications were performed blinded to group allocation, and the average value per mouse was used as one biological replicate for statistical analyses.

### 2.11 RNA isolation and quantitative real-time PCR

Quantitative reverse-transcription PCR (qRT-PCR) was performed as previously described. Total RNA was isolated with TRIzol reagent (Life Technologies, Grand Island, NY) according to the manufacturer’s instructions. Two micrograms of RNA were reverse-transcribed using the PrimeScript RT kit (Vazyme, R323-01), and amplification was carried out with SYBR Green PCR Master Mix (Vazyme, Q341-02) on a StepOnePlus Real-Time PCR System (Applied Biosystems, USA). *Gapdh* was used as an endogenous control. Primer sequences are provided in **Supplementary Table S5**.

### 2.12 Statistical analysis

Quantitative data are presented as mean ± SD. Figures were generated in GraphPad Prism 9 (GraphPad Software, CA, USA), and statistical analyses were performed in SPSS 23.0 (SPSS Inc., Chicago, USA). Two-group comparisons were performed using unpaired, two-tailed Student’s t tests, and comparisons among three or more groups used one-way ANOVA with appropriate *post hoc* multiple-comparison procedures. A two-sided p< 0.05 was considered statistically significant.

## 3 Result

### 3.1 Integrated transcriptomics reveals immune–metabolic dysregulation in renal allograft fibrosis

To investigate transcriptional features associated with renal allograft fibrosis, we integrated three GEO datasets (GSE44131, GSE53605 and GSE76882) and performed batch effect correction using the SVA algorithm. Principal component analysis (PCA) and boxplot analyses confirmed effective removal of batch effects: samples from different datasets were clearly separated before correction but became well mixed afterward, with comparable normalized gene expression distributions across datasets (**Figures 2A, B**; **Supplementary Figures S1A, B**).

Differential expression analysis between fibrotic (IFTA) and non-fibrotic allografts identified 1,304 DEGs, including 671 upregulated and 633 downregulated genes (**Figure 2C**). Pathway enrichment analyses revealed that these DEGs were significantly involved in both metabolic and immune processes. KEGG analysis indicated the enrichment in cytokine–cytokine receptor interaction, NF-κB signaling, T cell differentiation, and carbon metabolism (**Figure 2D**), while Reactome and GO analyses further highlighted neutrophil degranulation, T cell activation, adaptive immune response, fatty acid metabolism, and extracellular matrix remodeling (**Supplementary Figures S1C, D**). These findings suggest that immune dysregulation and metabolic reprogramming jointly contribute to the progression of allograft fibrosis. To assess the robustness of our findings, we performed sensitivity analyses using more stringent DEG thresholds (|log2FC| > 0.75 with P< 0.05). Although the exact gene lists varied, the major enriched pathways and biological themes remained highly consistent across these settings (**Supplementary Figures S2A–G**).

Given the critical role of lactylation in orchestrating metabolic and immune regulation, we intersected the DEGs with a lactylation-related gene set and identified 72 differentially expressed lactylation-related genes (DELRGs) (**Figure 2E**). Pathway and process enrichment analysis of these DELRGs was performed using Metascape (http://metascape.org), followed by construction of pathway clusters and functional interaction networks based on gene set overlaps. The analysis revealed significant enrichment of lactate metabolism, myeloid cell differentiation, and lymphocyte activation (**Figure 2F**). Network mapping further delineated tightly interconnected modules centered on lactate metabolism and immune activation, supporting a lactate-driven metabolic–immune axis as a critical contributor to the pathogenesis of renal allograft fibrosis (**Figure 2G**).

### 3.2 Identification of key co-expression module and fibrosis-related lactylation genes via WGCNA

**Figure 3:**
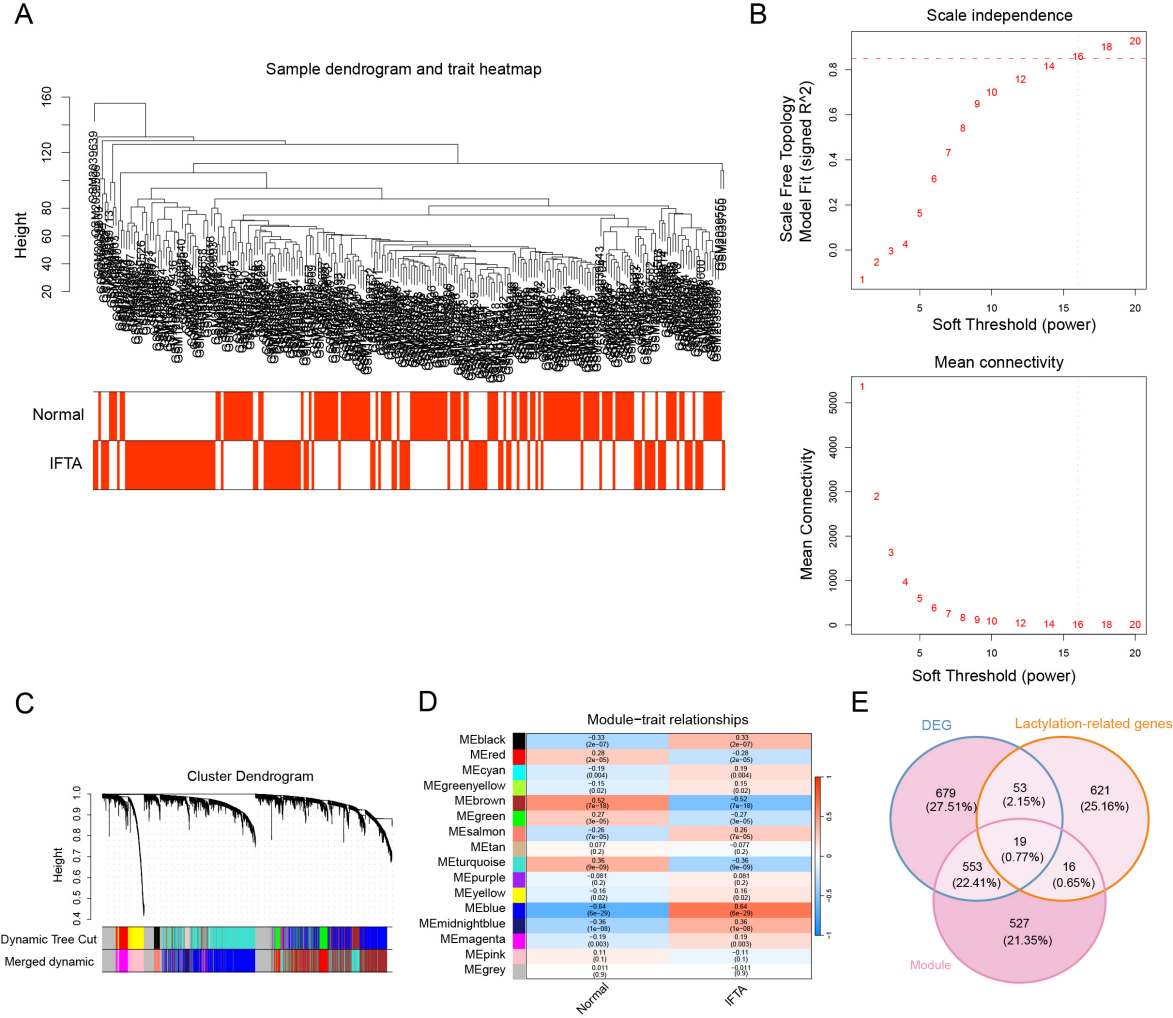
Identification of key co-expression module and fibrosis-related lactylation genes via WGCNA. **(A)** Sample clustering dendrogram to detect and remove outlier samples. **(B)** Analysis of scale independence and mean connectivity to determine the optimal soft-thresholding power (β = 16, R² > 0.85). **(C)** Cluster dendrogram of genes and module assignment by dynamic tree cutting. **(D)** Heatmap of module–trait correlations showing that the turquoise module was most strongly associated with IFTA. **(E)** Venn diagram displaying the overlap among blue module genes, DEGs, and lactylation-related genes (LRGs).

To further explore gene networks underlying renal allograft fibrosis, we performed weighted gene co-expression network analysis (WGCNA). All samples were hierarchically clustered, and outliers were removed based on sample dendrograms (**Figure 3A**). We then determined the optimal soft-thresholding power as β = 16, which achieved approximate scale-free topology with R² > 0.85 and ensured reliable network construction (**Figure 3B**).

Using dynamic tree cutting and a minimum module size of 50, a total of 16 co-expression modules were identified (**Figure 3C**). We next correlated module eigengenes with clinical traits (Normal vs IFTA). Among these, the blue module showed the strongest positive correlation with IFTA status (R = 0.64, P< 0.001), suggesting its potential role in fibrosis (**Figure 3D**). To assess the robustness of the WGCNA modules, we performed module preservation analysis using the merged network as the reference and each individual dataset (GSE44131, GSE53605 and GSE76882) in turn as the test set. Most modules showed strong preservation with high Zsummary values, and the blue module in particular displayed consistently high preservation statistics (Zsummary > 10) across all three datasets (**Supplementary Figures S3A–C**), indicating that its co-expression structure is reproducible despite cohort and platform differences.

Finally, we intersected genes from the blue module, DEGs and LRGs. This analysis yielded 19 overlapping genes (**Figure 3E**), which were regarded as candidate fibrosis-related lactylation genes for subsequent analyses.

### 3.3 Machine learning–based identification of hub lactylation-related genes in renal allograft fibrosis

After identifying fibrosis-associated co-expression modules using WGCNA, we applied five complementary machine-learning algorithms—Random Forest (RF), LASSO, Support Vector Machine–Recursive Feature Elimination (SVM-RFE), XGBoost, and ElasticNet—to systematically prioritize lactylation-related genes with the highest predictive potential. This integrative approach complements network-based analysis by refining hub gene selection at the single-gene level, thereby enhancing both model robustness and biological interpretability.

**Figure 4:**
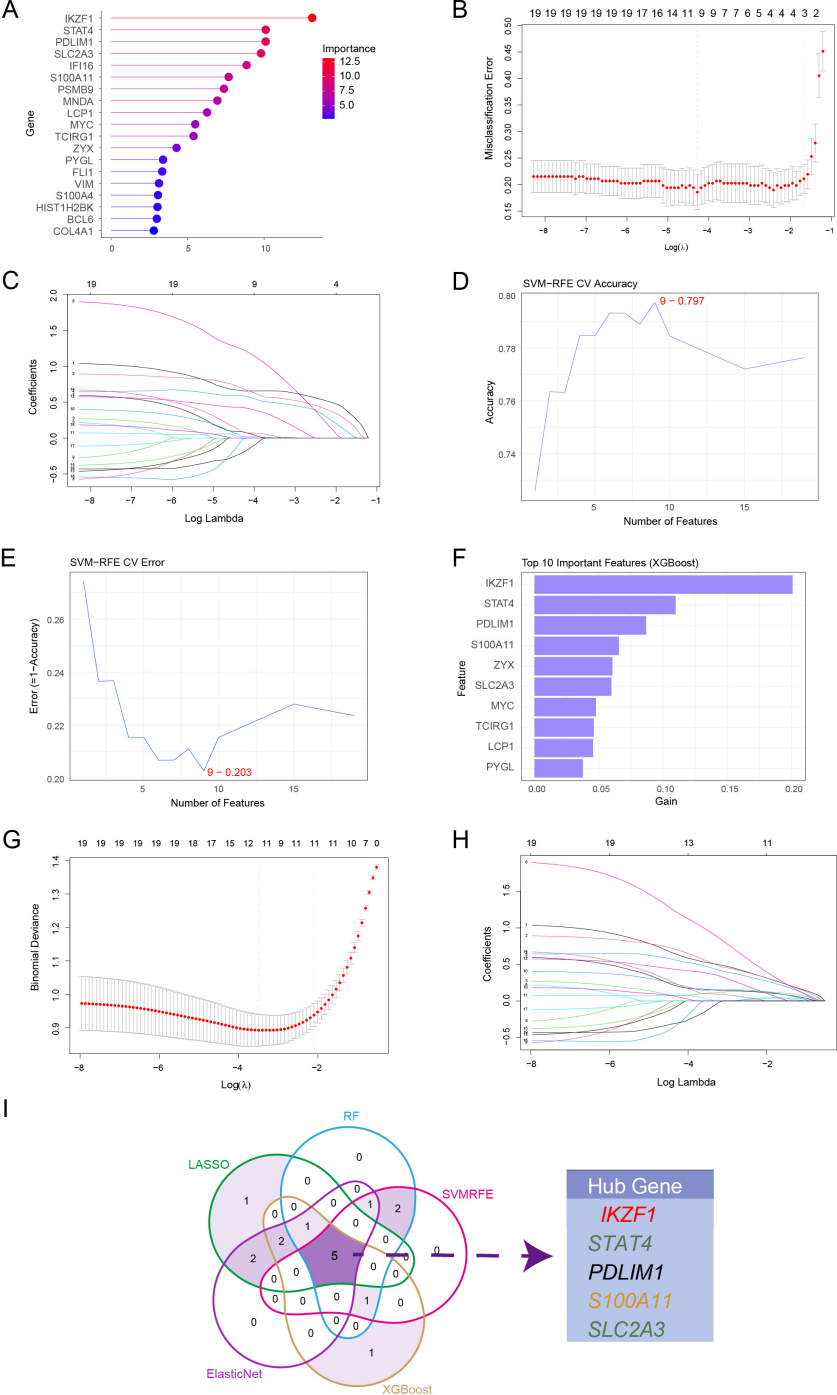
Machine learning–based identification of hub lactylation-related genes in renal allograft fibrosis. **(A)** Random Forest (RF) ranking of feature importance. **(B, C)** LASSO regression analysis minimizing misclassification error and selecting optimal features. **(D, E)** SVM-RFE cross-validation showing maximal accuracy and minimal error when nine features were retained. **(F)** Top 10 important features identified by XGBoost. **(G, H)** ElasticNet regression analysis for feature selection under L1/L2 regularization. **(I)** Venn diagram displaying overlap of selected features across five algorithms, identifying five consistent hub genes (*IKZF1, STAT4, PDLIM1, S100A11* and *SLC2A3*).

Random Forest analysis highlighted *IKZF1, STAT4, PDLIM1, SLC2A3*, and *S100A11* as the top-ranked genes in importance scoring (**Figure 4A**). LASSO regression minimized classification error and binomial deviance, retaining a parsimonious subset of predictive features (**Figures 4B, C**). SVM-RFE further optimized gene selection, achieving the highest classification accuracy and lowest error when nine features were included (**Figures 4D, E**). XGBoost consistently identified *IKZF1, STAT4, PDLIM1*, and *S100A11* as critical contributors to model performance (**Figure 4F**). ElasticNet regression provided stable feature selection under combined L1 and L2 regularization (**Figures 4G, H**).

By intersecting the top-ranked features across all five algorithms, we identified five robust hub genes—*IKZF1, STAT4, PDLIM1, S100A11*, and *SLC2A3*—that were consistently retained (**Figure 4I**). These genes were selected as core candidates for further validation and construction of the lactylation-related risk score model.

### 3.4 Clinical correlation between hub lactylation-related genes and renal function

We further investigated the clinical relevance of the five hub lactylation-related genes in relation to renal function. Correlation analyses in the Nephroseq v5 dataset revealed that *STAT4, PDLIM1, IKZF1*, and *S100A11* expression levels were strongly and significantly associated with kidney function parameters. Specifically, higher expression of these genes correlated negatively with estimated glomerular filtration rate (eGFR) and positively with serum creatinine, indicating their close link with renal impairment (**Supplementary Figures S4A–D**). Among them, *IKZF1* showed the strongest correlations (R² = 0.37 with eGFR and R² = 0.32 with serum creatinine), underscoring its potential as a robust biomarker. *SLC2A3* displayed weaker correlations but exhibited consistent trends (**Supplementary Figure S4E**). Collectively, these results highlight that lactylation-related hub genes are closely related to renal dysfunction and may serve as diagnostic and prognostic markers in renal allograft fibrosis.

### 3.5 Construction and validation of a lactylation-related risk score for renal allograft fibrosis

**Figure 5:**
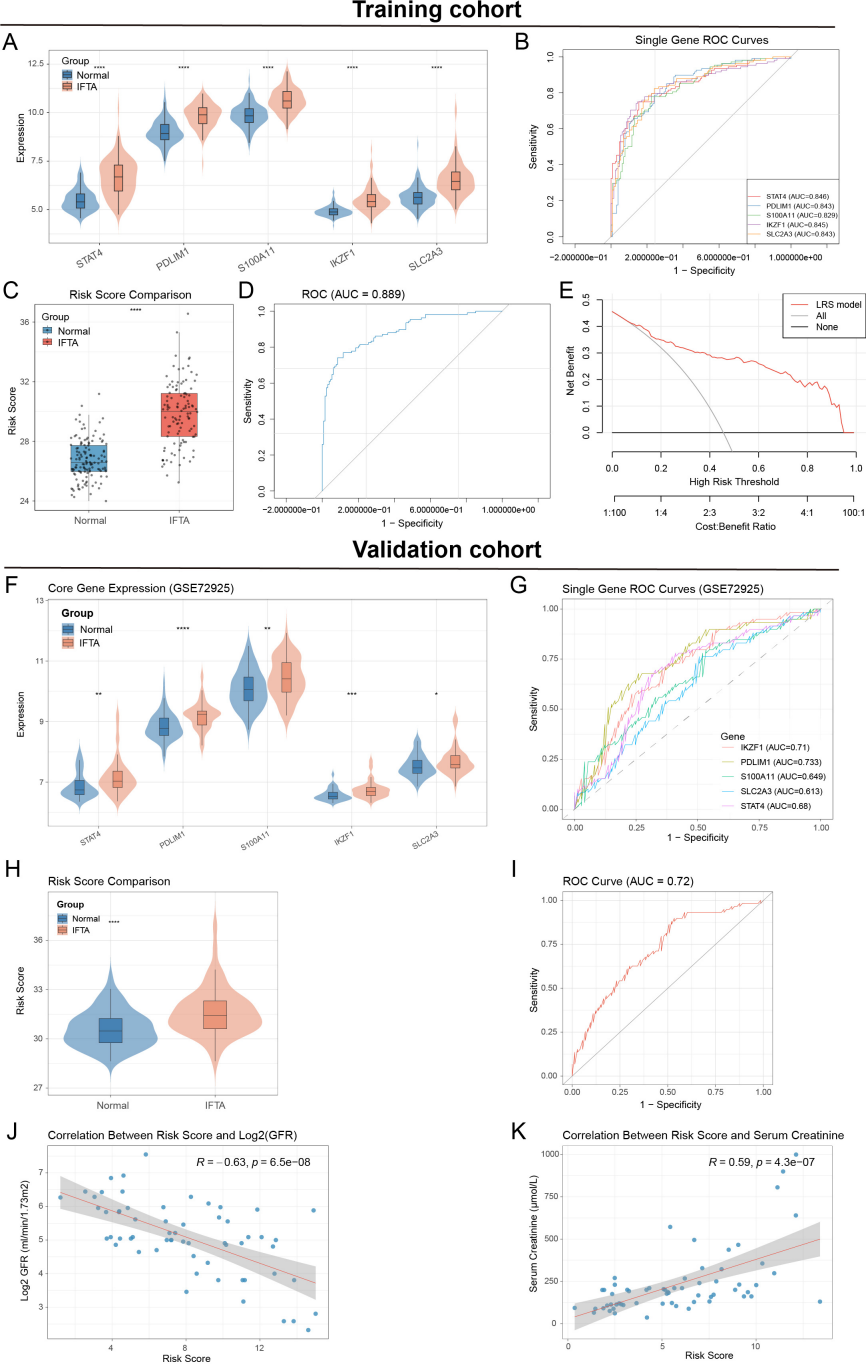
Construction and validation of a lactylation-related risk score for renal allograft fibrosis. **(A)** Violin plots showing expression levels of five hub genes (*IKZF1, STAT4, PDLIM1, S100A11* and *SLC2A3*) in normal vs. IFTA samples. **(B)** ROC curves of individual genes in the training set. **(C)** Comparison of risk scores between normal and IFTA samples. **(D)** Combined ROC analysis of the integrated risk score in the training set. **(E)** Decision curve analysis of the lactylation-related risk score (LRS) in the training cohort, comparing the net benefit of the LRS model (red) with treat-all (grey) and treat-none (black) strategies across threshold probabilities. **(F)** Expression validation of hub genes in the external dataset GSE72925. **(G)** ROC curves of individual genes in the validation set. **(H)** Comparison of risk scores in validation samples. **(I)** ROC curve of the integrated risk score in the validation set. **(J)** Correlation between risk score and eGFR. **(K)** Correlation between risk score and serum creatinine. *p< 0.05, **p< 0.01, ***p< 0.001, ****p< 0.0001.

To evaluate the diagnostic value of the five hub lactylation-related genes, we first examined their expression in normal and IFTA kidney allografts. All five genes (*STAT4, PDLIM1, S100A11*, *IKZF1*, and *SLC2A3*) were significantly upregulated in IFTA samples (**Figure 5A**). ROC curve analysis demonstrated good predictive accuracy for individual genes, with AUC values ranging from 0.82 to 0.84 (**Figure 5B**). We next integrated these hub genes to construct a lactylation-related risk score. The calculated risk score was significantly elevated in IFTA samples compared with controls (**Figure 5C**), and the combined ROC analysis yielded an AUC of 0.889, indicating strong diagnostic performance (**Figure 5D**). In the training cohort, calibration analysis demonstrated good agreement between LRS-predicted probabilities and the observed frequency of IFTA, and decision-curve analysis indicated that using the LRS would provide greater net clinical benefit than either a “treat-all” or “treat-none” strategy across a broad range of threshold probabilities (**Figure 5E**; **Supplementary Figure S6A**). The corresponding accuracy, sensitivity, and specificity for the training cohort are summarized in **Supplementary Table S3**.

The robustness of this risk score was validated in an independent cohort (GSE72925). Consistent with the training dataset, all five hub genes were significantly upregulated in IFTA samples (**Figure 5F**), and individual ROC curves again showed moderate diagnostic power (AUC values 0.61–0.73; **Figure 5G**). Importantly, the risk score remained significantly higher in IFTA patients (**Figure 5H**), and the integrated ROC curve confirmed acceptable diagnostic accuracy (AUC = 0.72, **Figure 5I**). In the validation cohort, the calibration curve showed generally acceptable agreement between predicted and observed risk, albeit with greater variability, and decision-curve analysis likewise supported a positive net benefit of the LRS compared with default “treat-all” or “treat-none” approaches (**Supplementary Figure S6B**). Finally, we explored the clinical relevance of the risk score. Correlation analysis revealed that higher risk scores were inversely associated with eGFR (R = −0.63, P = 6.5×10^-8^; **Figure 5J**) and positively correlated with serum creatinine levels (R = 0.59, P = 4.3×10^-7^, **Figure 5K**), suggesting that the lactylation-related signature reflects renal function decline.

### 3.6 Metabolic rewiring and immune activation characterize high-LRS IFTA

Next, we profiled the transcriptomic landscape of the high-LRS IFTA subgroup. Using the lactylation-related risk score (LRS), IFTA samples were stratified into high- and low-risk groups (**Supplementary Figure S7A**). Differential expression analysis between high- and low-risk groups identified 1,664 DEGs, including 901 upregulated and 763 downregulated genes (**Supplementary Figure S7B**).

**Figure 6:**
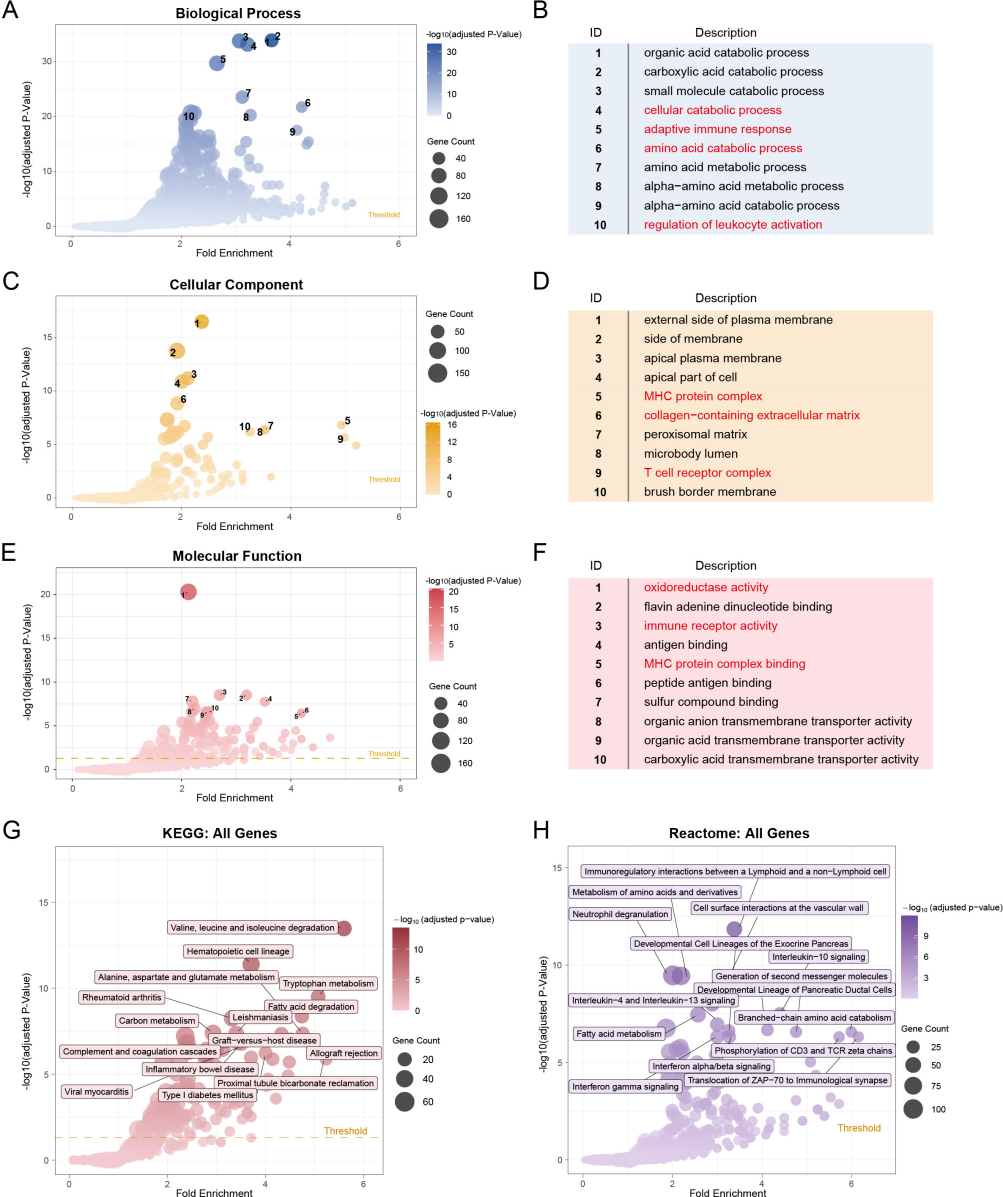
Metabolic rewiring and immune activation characterize high-LRS IFTA. **(A, B)** GO Biological Process (BP) enrichment showing terms related to metabolism and immunity. **(C, D)** GO Cellular Component (CC) enrichment highlighting immune-related components and fibrosis-related structures. **(E, F)** GO Molecular Function (MF) enrichment including oxidoreductase activity, immune receptor activity, and MHC protein complex binding. **(G)** KEGG pathway enrichment analysis of lactylation-related genes. **(H)** Reactome pathway enrichment analysis showing immune– metabolic crosstalk.

GO enrichment analysis showed that metabolic and immune-related terms dominated in the high-risk group. In Biological Processes (BP), genes were enriched in cellular catabolic processes and amino acid catabolic processes, indicating metabolic reprogramming, as well as immune-related processes such as adaptive immune response and regulation of leukocyte activation (**Figures 6A, B**). For Cellular Components (CC), enrichment was observed in the MHC protein complex, T-cell receptor complex, and collagen-containing extracellular matrix (**Figures 6C, D**). Molecular Functions (MF) were characterized by oxidoreductase activity, immune receptor activity, and MHC protein complex binding (**Figures 6E, F**). Pathway analysis further confirmed this immunometabolic profile: KEGG pathways were enriched for valine, leucine and isoleucine degradation, carbon metabolism, and allograft rejection (**Figure 6G**), while Reactome terms included immunoregulatory interactions between lymphoid and non-lymphoid cells, neutrophil degranulation, and biological oxidations (**Figure 6H**). Overall, the high-risk group is characterized by metabolic reprogramming, immune activation, and extracellular matrix remodeling compared to the low-risk group.

### 3.7 Immune landscape of IFTA and lactylation risk-stratified groups

**Figure 7:**
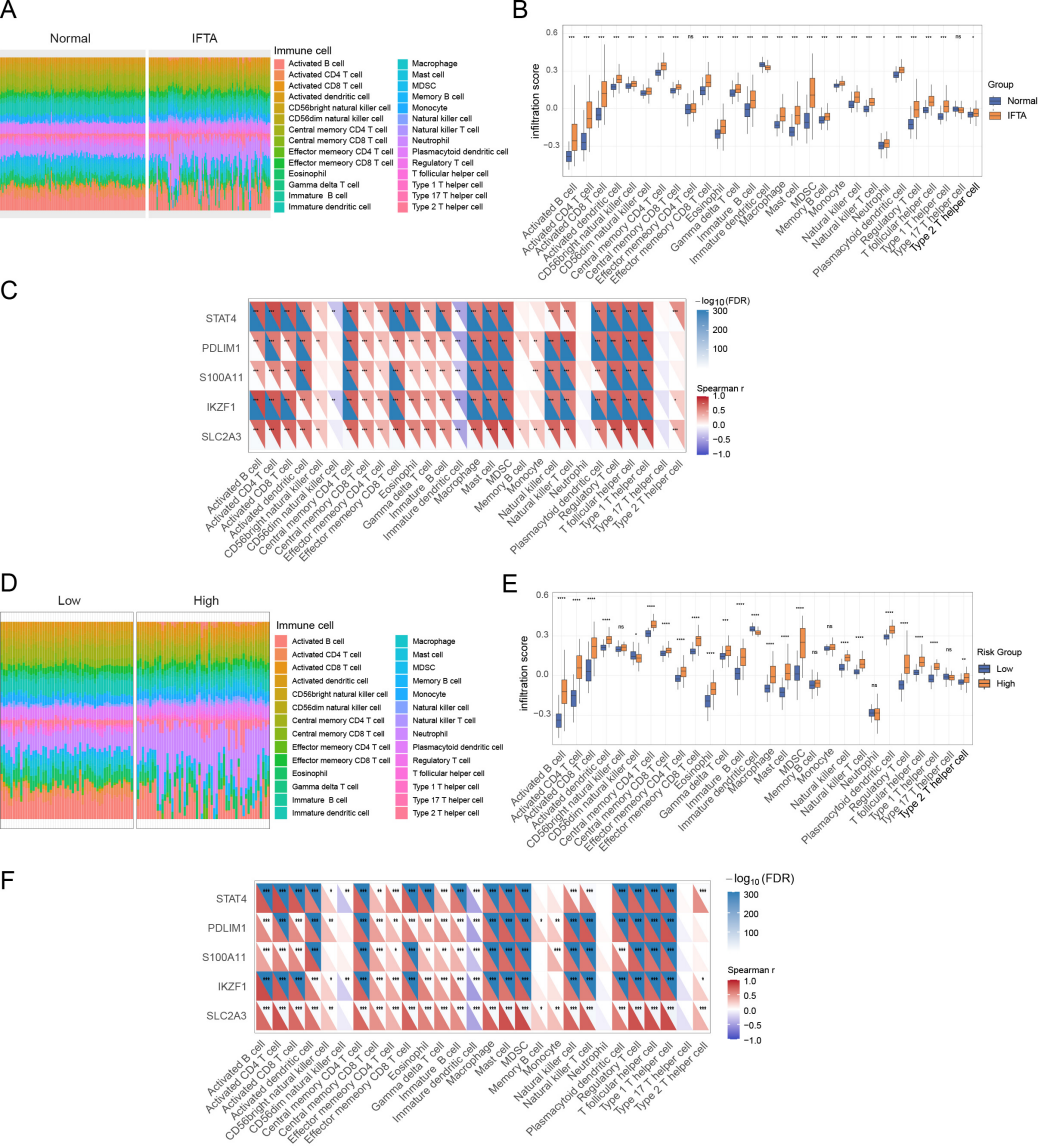
Immune landscape of IFTA and lactylation risk-stratified groups. **(A)** Stacked barplot showing the relative proportions of 28 immune cell types in normal and IFTA samples. **(B)** Boxplot comparing infiltration scores of immune cell subsets between normal and IFTA groups. **(C)** Correlation heatmap between hub lactylation-related genes (*STAT4, PDLIM1, S100A11, IKZF1* and *SLC2A3*) and immune cell infiltration across samples. **(D)** Stacked barplot showing the immune infiltration landscape stratified by lactylation-related risk score (low vs. high). **(E)** Boxplot showing differences in immune infiltration scores between high- and low-risk groups. **(F)** Correlation heatmap illustrating the associations between hub lactylation-related genes and immune cells in high- vs. low-risk groups. *p< 0.05, **p< 0.01, ***p< 0.001.

Given the established role of aberrant immune responses and chronic inflammation in post-transplant renal fibrosis (44), we used ssGSEA to characterize immune cell infiltration in IFTA samples. Comparison with normal allografts revealed a distinct immune profile, with IFTA tissues showing markedly higher levels of proinflammatory immune subsets, including activated CD4^+^ and CD8^+^ T cells, γδ T cells, NK cells, and macrophages (**Figures 7A, B**). Pairwise immune–immune correlation matrices supported this pattern: relative to normal allografts, IFTA showed tighter positive coupling among effector/innate inflammatory compartments and reduced links with regulatory subsets (**Supplementary Figure S8A**). Correlation analysis demonstrated strong associations between the expression of hub lactylation-related genes (*STAT4, PDLIM1, S100A11, IKZF1*, and *SLC2A3*) and these proinflammatory immune populations (**Figure 7C**). Stratification by the lactylation-related risk score (LRS) further showed that high-risk patients exhibited significantly increased infiltration of macrophages, dendritic cells, and effector T cell subsets compared with low-risk individuals (**Figures 7D, E**). Consistently, the high-LRS correlation network mirrored the IFTA pattern, whereas the low-LRS network resembled normal (**Supplementary Figure S8B**). Heatmap correlations showed that all five hub genes were positively associated with proinflammatory immune cells in both groups, linking lactylation-related genes to immune-driven fibrosis (**Figure 7F**).

### 3.8 snRNA-seq reveals expanded immune infiltration, elevated lactylation, and cell-type–specific LRG patterns in IFTA

**Figure 8:**
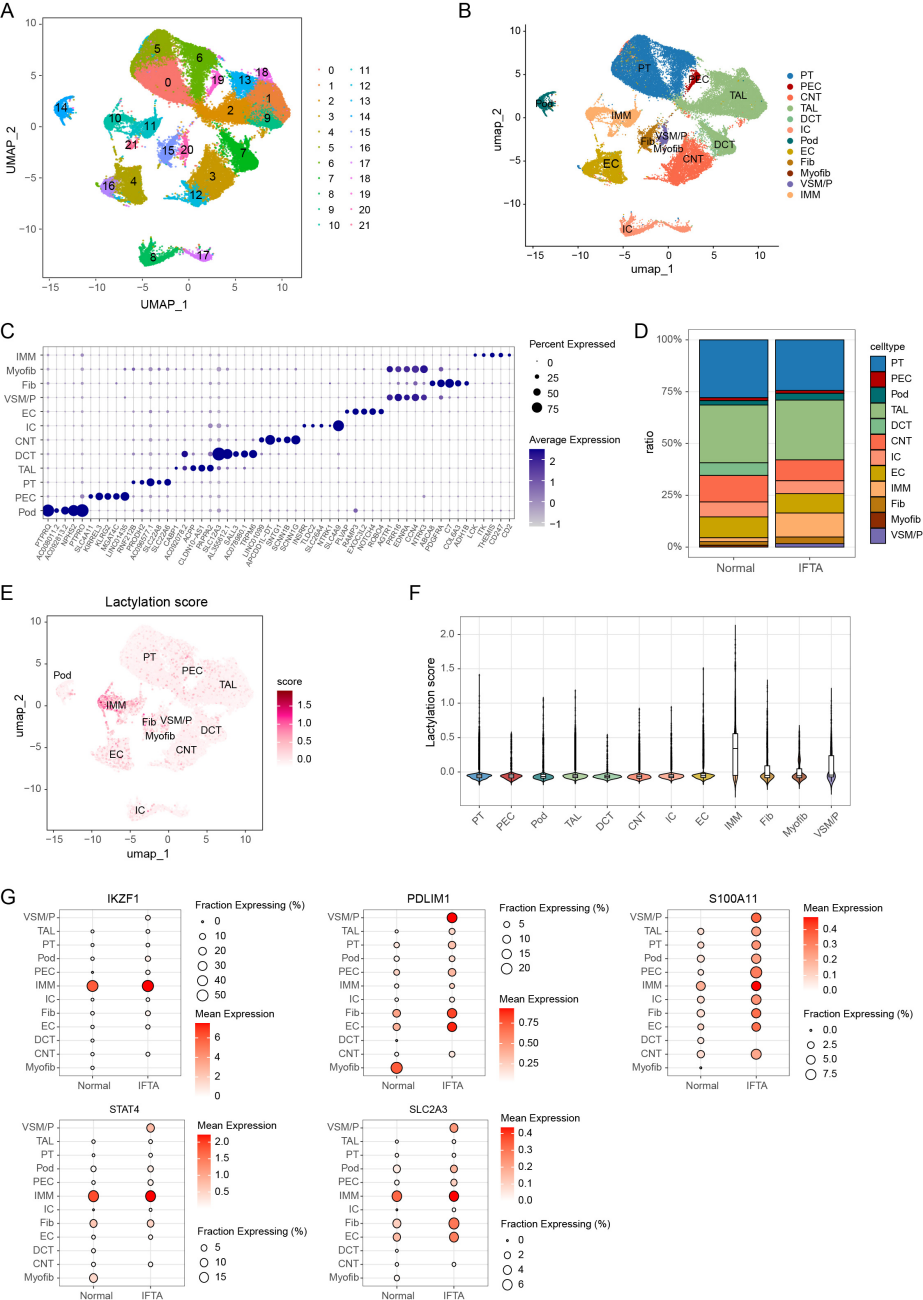
snRNA-seq reveals expanded immune infiltration, elevated lactylation, and cell-type–specific LRG patterns in IFTA. **(A)** Harmony-integrated UMAP of GSE195718 showing 21 clusters from six IFTA and three normal/nonspecific allograft biopsies. **(B)** UMAP after manual annotation and merging into 12 cell classes by canonical markers. **(C)** Bubble plot of the marker genes used for annotation; dot size shows the fraction of nuclei expressing the marker, color indicates average expression. **(D)** Stacked bars of cell-type composition by condition. **(E)** Feature map of the lactylation module score (Seurat module scoring) projected onto the UMAP. **(F)** Violin plots of lactylation scores across the 12 cell classes. **(G)** Dot plots for the five hub lactylation-related genes (LRGs)—*IKZF1, STAT4, PDLIM1, SLC2A3, S100A11*—stratified by condition (IFTA vs Normal). Dot size denotes the fraction expressing and color indicates mean expression. PT, proximal tubule; PEC, parietal epithelial cell; CNT, connecting tubule; TAL, thick ascending limb; DCT, distal convoluted tubule; IC, intercalated cell; POD, podocyte; EC, endothelial cell; Fib, fibroblast; Myofib, myofibroblast; VSM/P, vascular smooth muscle/pericyte; IMM, immune cell.

Building on the immune-infiltration patterns above, we next analyzed the snRNA-seq dataset GSE195718 (six biopsies from chronic allograft dysfunction with IFTA and three from patients with stable graft function and normal or nonspecific histology) to map these changes to specific cell types. After integration and batch correction, a UMAP revealed 21 clusters (**Figure 8A**). Using canonical markers (bubble plot), these clusters were merged into 12 cell classes: proximal tubule (PT), parietal epithelial cell (PEC), connecting tubule (CNT), thick ascending limb (TAL), distal convoluted tubule (DCT), intercalated cell (IC), podocyte (POD), endothelial cell (EC), fibroblast (Fib), myofibroblast (Myofib), vascular smooth muscle/pericyte (VSM/P), and immune cell (IMM) (**Figures 8B, C**). Consistent with our immune-infiltration analysis, cell-composition profiling (stacked bars) showed a marked increase in immune cells in IFTA compared with Normal (**Figure 8D**). To formally quantify these differences, we compared sample-level cell fractions between groups and found that IFTA biopsies had significantly higher proportions of immune cells, fibroblasts and myofibroblasts, along with reduced fractions of PT, TAL and other tubular epithelial cells (**Supplementary Figure S9D**).

Module scoring of a lactylation-related gene set (feature map and violin) indicated that the lactylation signature was elevated in IFTA and skewed toward immune lineages, with lower scores in most epithelial and endothelial populations (**Figures 8E, F**; **Supplementary Figure S9C**). Consistent with the bulk-transcriptomic results, all five hub lactylation-related genes (LRGs) were upregulated in IFTA and showed distinct cell type–specific expression patterns. Specifically, IKZF1 and STAT4 were predominantly expressed in immune-cell clusters with only minimal signals in parenchymal lineages; PDLIM1 was enriched in interstitial stromal populations, particularly myofibroblasts and vascular smooth muscle/endothelial cells; SLC2A3 was mainly detected in infiltrating immune cells and fibroblasts; and S100A11 was broadly induced across multiple cell types in IFTA, including tubular epithelial, endothelial and immune cells (**Figure 8G**; **Supplementary Figures S9A, B**). Together, these findings validate increased immune infiltration and immune-skewed lactylation in IFTA and delineate unique LRG programs at single-cell resolution.

### 3.9 Experimental validation of lactylation-related hub genes in murine IRI and UUO models

**Figure 9:**
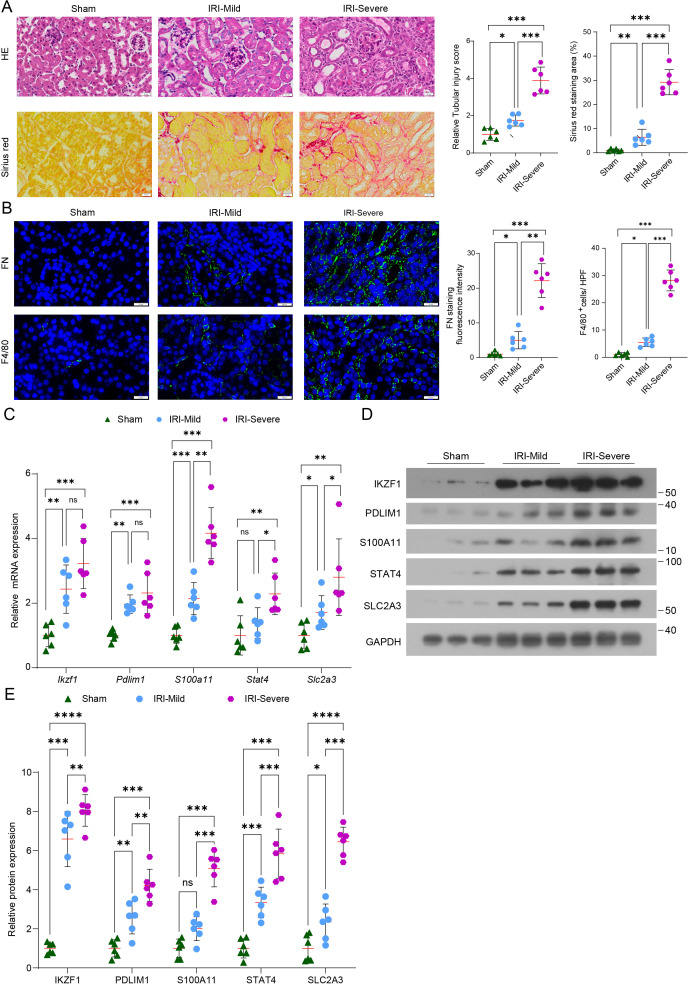
Experimental validation of lactylation-related hub genes in a murine IRI model. **(A)** Representative H&E and Sirius Red staining of kidneys from Sham, IRI-Mild, and IRI-Severe groups, with quantification of tubular injury scores and collagen deposition. For each group, 5–6 mice were analyzed; for each mouse kidney, 10 randomly selected nonoverlapping high-power fields were quantified, and the average per mouse was used for statistics. **(B)** Immunofluorescence of fibronectin (FN) and F4/80 showing progressive extracellular matrix accumulation and macrophage infiltration; quantification on the right. **(C)** qPCR analysis demonstrating increased expression of *Ikzf1, Pdlim1, S100a11, Stat4*, and *Slc2a3* in IRI kidneys. **(D)** Representative Western blots showing protein levels of IKZF1, PDLIM1, S100A11, STAT4, and SLC2A3 in Sham, IRI-Mild, and IRI-Severe kidneys. **(E)** Densitometric quantification of Western blot signals normalized to GAPDH. Each dot represents one mouse. Data are mean ± SD; *p< 0.05, **p< 0.01, ***p< 0.001; ****p< 0.0001; ns, not significant. Scale bars: 20 μm.

To validate the findings from our bioinformatics analyses, we employed a graded renal ischemia–reperfusion injury (IRI) model to recapitulate progressive fibrosis and immune activation. Histopathological evaluation demonstrated a severity-dependent increase in tubular injury and collagen deposition. H&E staining revealed prominent tubular epithelial damage, tubular dilation, and inflammatory infiltration, with higher tubular injury scores in IRI-Mild and IRI-Severe mice compared with sham controls. Sirius Red staining confirmed marked interstitial collagen accumulation, highlighting extensive extracellular matrix remodeling (**Figure 9A**). Immunofluorescence for fibronectin (FN) and F4/80 showed enhanced FN deposition and a significant influx of F4/80^+^ macrophages in the IRI groups, particularly in severe injury kidneys, reflecting active fibrotic and inflammatory responses (**Figure 9B**). Quantitative PCR further verified upregulation of the five lactylation-related hub genes (*Ikzf1, Pdlim1, S100a11, Stat4 and Slc2a3*) in fibrotic kidneys, with expression levels increasing in parallel with injury severity (**Figure 9C**). Collectively, these experimental results corroborate the computational predictions.

To further validate the robustness of these hub genes across fibrosis settings, we independently examined their expression in our established UUO model. Consistent with the IRI findings, UUO kidneys exhibited significant mRNA upregulation of Ikzf1, Pdlim1, S100a11, Stat4, and Slc2a3 compared with sham controls (**Supplementary Figure S10C**). Moreover, Western blotting in UUO and IRI kidneys further confirmed increased protein abundance of these five hub genes, supporting their stable association with renal fibrogenesis *in vivo* (**Figures 9D, E**; **Supplementary Figures S10A, B**). Collectively, these experimental results support the findings from our bioinformatic analyses.

## 4 Discussion

Over-activated immune responses critically contribute to chronic allograft injury and progressive fibrosis (45). Long-term graft survival therefore remains a major clinical challenge (46). The causes of immune cells activation are diverse, but primarily from the pro-inflammatory environment (10). In renal allograft fibrosis, hypoxia and mitochondrial dysfunction in tubular epithelial cells promote a metabolic shift toward glycolysis, leading to excessive lactate production, which in turn strongly stimulates proinflammatory macrophage activation. In addition, excessive lactate could modify proteins in post-translational stage to form lactylation, which is recently found to be pivotal in fibrogenesis across multiple organs (47, 48).

Growing evidence indicates that lactylation marks are enriched in immune cells such as macrophages and T cells (49), suggesting a crucial role of lactylation in allograft fibrosis, where macrophages and T cells are persistently overactivated. Based on these insights, we focused on identifying key lactylation-related genes involved in renal allograft fibrosis.

In this study, we integrated multiple GEO datasets to identify lactylation-related genes associated with renal allograft fibrosis. Multiple enrichment analyses revealed that immune activation, oxidative stress and metabolic reprogramming—particularly lactylation-related pathways—are strongly implicated. Consistent with these findings, previous studies have shown that lactylation of H3K18 and H3K9 enhances CD8^+^ T-cell activation (50) and regulates macrophages (51), further suggesting a key role of lactylation in shaping immune responses.

We further identified five hub lactylation-related genes—*IKZF1, PDLIM1, S100A11, STAT4* and *SLC2A3*—that were strongly associated with impaired kidney function. By integrating WGCNA with multiple machine-learning approaches, we confirmed their close relationship with renal allograft fibrosis. We further performed ROC analysis and demonstrated their robust performance in diagnosis, suggesting their potential as predictive biomarkers. Building on these findings, we constructed a lactylation-related risk score (LRS) based on the five hub genes, which showed strong discrimination in the training cohort (AUC = 0.889) and retained acceptable diagnostic performance in the independent validation cohort GSE72925 (AUC = 0.72). Notably, when we stratified the training dataset GSE76882 by histologic subtypes, the LRS consistently achieved high, albeit slightly different, AUCs for distinguishing stable grafts (TX) from classic IFTA, IFTA with acute rejection (IFTA_AR), and IFTA with interstitial inflammation (IFTA_i), indicating that the score performs particularly well in fibro-inflammatory IFTA phenotypes (**Supplementary Figure S5A**). By contrast, the validation cohort included fewer samples, lacked detailed information on inflammatory subtypes, and was generated on a different microarray platform. Thus, the modest reduction in AUC in GSE72925 likely reflects a combination of sample heterogeneity, platform effects and unmeasured biological variability.

Among these hub genes, *IKZF1* encodes the transcription factor IKAROS (51). By enhancing antigen presentation and driving cytokine production in dendritic cells (DCs), *IKZF1* contributes to renal fibrosis and IgA nephropathy (52). *PDLIM1* (also known as CLP36, CLIM1, or Elfin) exerts multifaceted functions in cytoskeletal organization, DNA damage repair, and organogenesis (53, 54). Of interest, PDLIM1 has been reported to promote fibrosis in liver (55). *S100A11*, a calcium-binding protein of the S100 family containing two EF-hand domains, functions as an intracellular calcium sensor. It has been shown to mediate aristolochic acid I (AA-I)- but not aristolochic acid IVa (AA-IVa)-induced renal interstitial fibrosis (RIF) (56). *STAT4* is a transcription factor mediating immune cell signal transduction and transcriptional activation (57). Previous studies showed *STAT4* is a core regulator of T cell function, which is controlled by PTPN6 and is closely linked to impaired allograft function and poor prognosis in renal transplantation (58). *SLC2A3* encodes the high-affinity glucose transporter GLUT-3, whose membrane translocation promotes glycolytic bursts and lactate accumulation (57). In fibrotic skin disease, activation of the TAGLN–RhoA/ROCK2–*SLC2A3* axis in fibroblasts couples mechanosensing of tissue stiffness to glycolytic reprogramming; however, whether this axis contributes to post-transplant renal fibrosis remains to be elucidated (59).

Aberrant immune responses and chronic inflammation play pivotal roles in post-transplant renal fibrosis (60–62). In our findings, we found the overactivated CD4^+^ and CD8^+^ T cells, γδ T cells, NK cells, dendritic cells, and macrophages in renal allograft fibrosis. Furthermore, lactylation were highly involved in these immune-cell activation. These findings suggest lactate is no longer a metabolite, but a key pathogenic factor, since it would lead to lactylation modifications through modifying lysine residues. Reports have shown lactylation highly contributes to immune diseases (63, 64). However, its role in renal allograft fibrosis has remained unclear.

Innovatively, we defined five lactylation-related hub genes—*IKZF1, PDLIM1, S100A11, STAT4* and *SLC2A3*. They were highly involved in renal allograft fibrosis through intimate associations with pro-inflammatory and profibrotic effects. In the broader context of chronic alloimmunity, epigenetic regulation—including DNA methylation and canonical histone modifications such as acetylation and methylation—has been increasingly linked to chronic graft dysfunction and fibrotic remodeling (10). As a lactate-derived lysine acylation mark occurring on histone lysine residues that are also frequent targets of classical modifications, histone lactylation adds a metabolite-coupled layer to this epigenetic landscape and may reshape transcriptional programs under persistent inflammatory stress (11). To our knowledge, this is the first study to systematically investigate lactylation-related genes in the setting of kidney transplantation. Compared with previous transcriptomic and integrative studies of renal allograft fibrosis that mainly focused on canonical inflammatory and profibrotic pathways (such as necroptosis, extracellular matrix genes and immune-related signatures) (30, 61, 65–67), our work specifically interrogates lactylation-related genes as a lactate-coupled epigenetic axis that bridges metabolic reprogramming and immune activation in chronic allograft injury, and integrates bulk transcriptomic cohorts, multiple machine-learning algorithms, single-nucleus RNA-seq data and *in vivo* fibrosis models within a single framework. This multi-layered design allows us not only to propose a mechanistically coherent immunometabolic LRG signature and to construct and externally validate an LRG-based diagnostic score that may complement existing histological and molecular classifiers, but also to reveal distinct pathway enrichment patterns and immune-cell infiltration profiles between high- and low-risk groups. Although this study still requires rigorous biological validation or clinical investigation, the results provide proof of principle that these LRGs and the LRG-based model may serve as novel diagnostic and therapeutic targets for renal allograft fibrosis.

While our findings provide new insights, several limitations should be acknowledged. First, the mechanistic roles of these genes in fibrosis progression and immune modulation remain to be elucidated, which will be a focus in our future studies. Second, similar to prior studies (65, 68), we chose a murine ischemia–reperfusion injury (IRI) model to validate fibrosis-related signatures. However, IRI primarily models ischemic tubular injury and subsequent fibrosis, and therefore only partially reflects the alloimmune context of chronic allograft fibrosis. In our human in silico analyses, allograft transcriptomes were characterized by fibrosis pathways (for example, extracellular matrix remodeling), and alloimmune-specific immune activation (such as antigen presentation and T-cell activation). We did not systematically quantify how strongly the human alloimmune pathways are recapitulated in IRI, which represents an additional limitation. Future studies incorporating human allograft biopsy samples and murine kidney transplant models will therefore be required to dissect shared versus alloimmune-specific pathways and to validate the functions of these genes in a truly alloimmune setting. Finally, although our diagnostic model was validated across multiple datasets, larger-scale, multi-center clinical trials are necessary to confirm its stability and applicability.

## 5 Conclusion

In summary, we identified five hub lactylation-related genes (LRGs)—*IKZF1, PDLIM1, S100A11, STAT4* and *SLC2A3*—that are closely associated with renal allograft fibrosis. Our findings underscore that lactylation represents an important immunometabolic layer in chronic allograft injury and suggest that LRGs and the LRG-based risk model merit further investigation as mechanistic drivers and potential diagnostic and therapeutic targets in renal allograft fibrosis.

## Data Availability

The datasets presented in this study can be found in online repositories. The names of the repository/repositories and accession number(s) can be found in the article/**Supplementary Material**.
