# Supplementary material for: Identification of lactylation and its hub genes in contributing immune activation and renal allograft fibrosis by integrative bioinformatics and machine learning
Source: Front Immunol. 2026 Feb 10;17:1741864. doi: 10.3389/fimmu.2026.1741864 (PMC12932934; doi:10.3389/fimmu.2026.1741864)
Supplement: Supplementary file 3 [file Presentation1.pptx]

## Slide 1
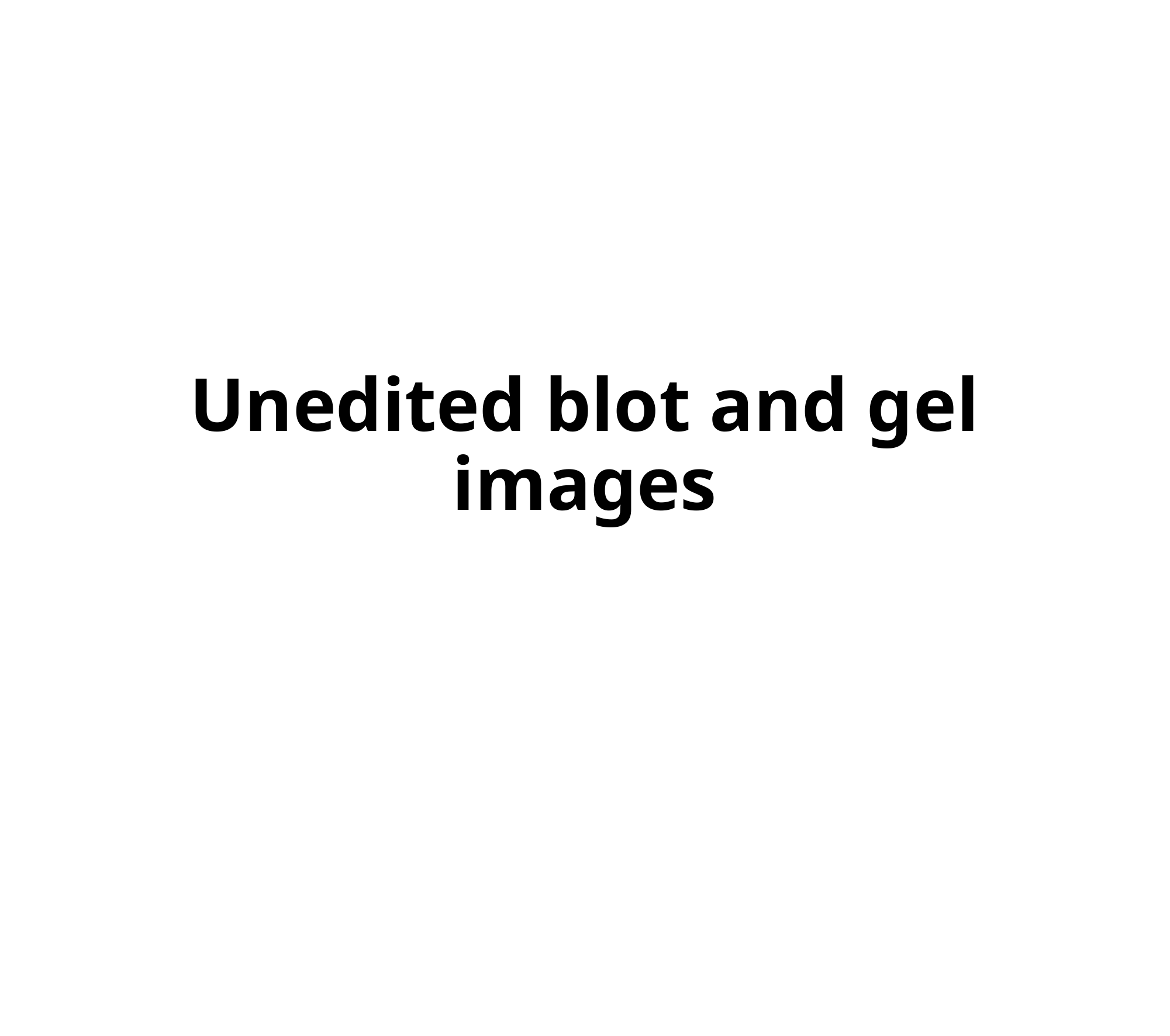

# Unedited blot and gel images

## Slide 2
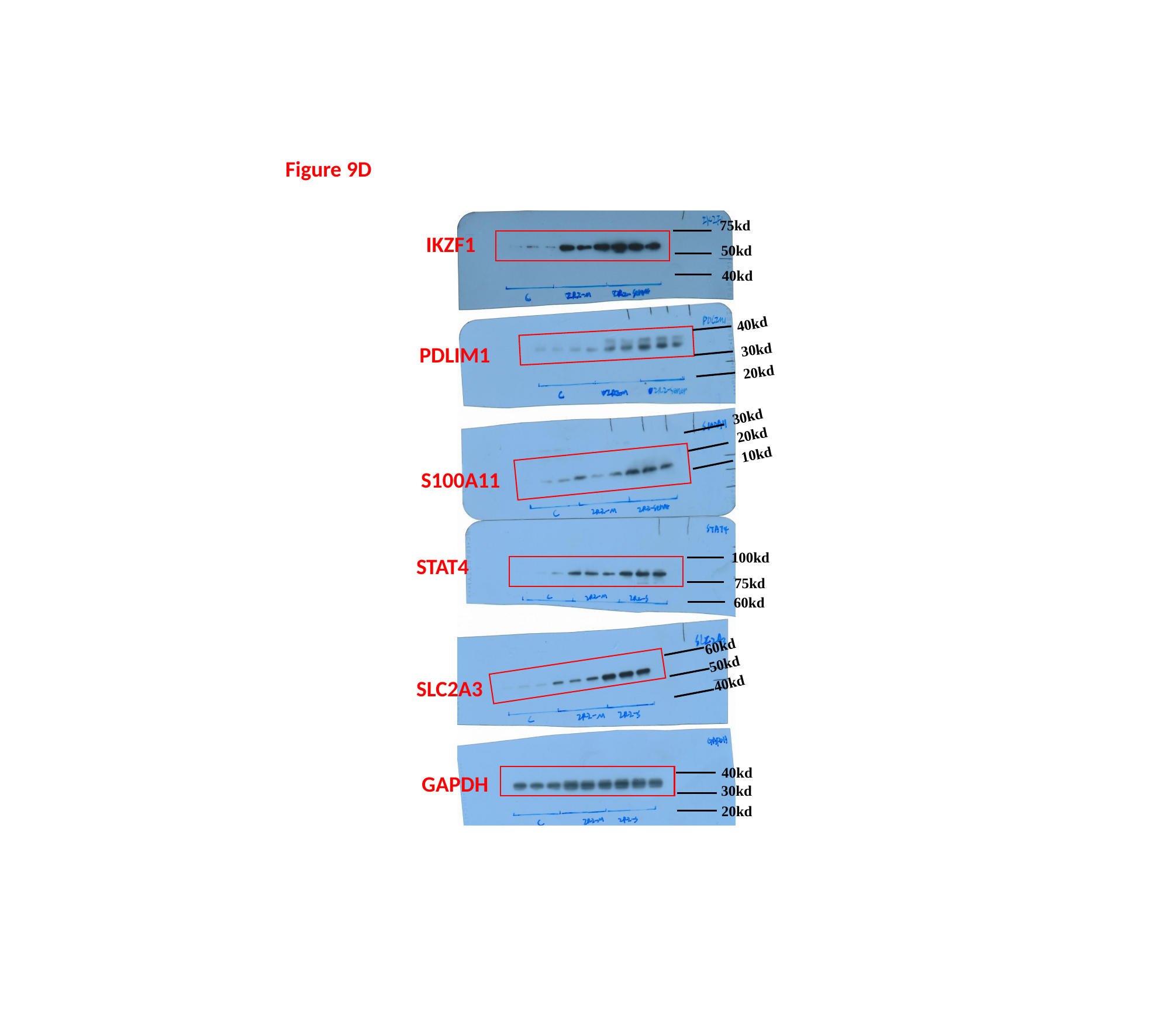

Figure 9D
75kd
IKZF1
50kd
40kd
40kd
30kd
PDLIM1
20kd
30kd
20kd
10kd
S100A11
100kd
STAT4
75kd
60kd
60kd
50kd
40kd
SLC2A3
40kd
GAPDH
30kd
20kd

## Slide 3
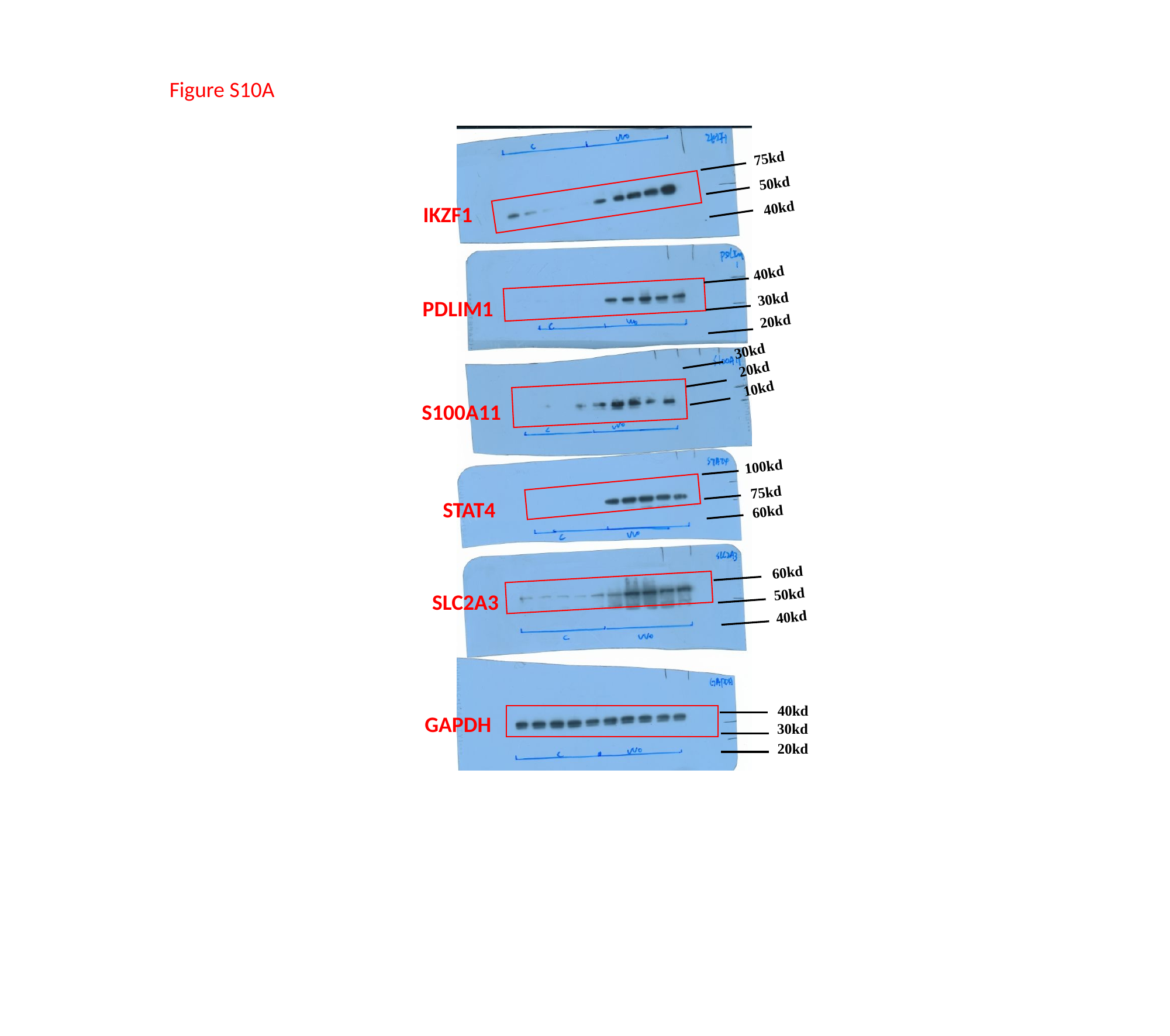

Figure S10A
75kd
50kd
40kd
IKZF1
40kd
30kd
20kd
PDLIM1
30kd
20kd
10kd
S100A11
100kd
75kd
60kd
STAT4
60kd
50kd
SLC2A3
40kd
40kd
30kd
20kd
GAPDH
