# Supplementary material for: Identification of lactylation and its hub genes in contributing immune activation and renal allograft fibrosis by integrative bioinformatics and machine learning
Source: Front Immunol. 2026 Feb 10;17:1741864. doi: 10.3389/fimmu.2026.1741864 (PMC12932934; doi:10.3389/fimmu.2026.1741864)
Supplement: Supplementary file 4 [file Table1.docx]

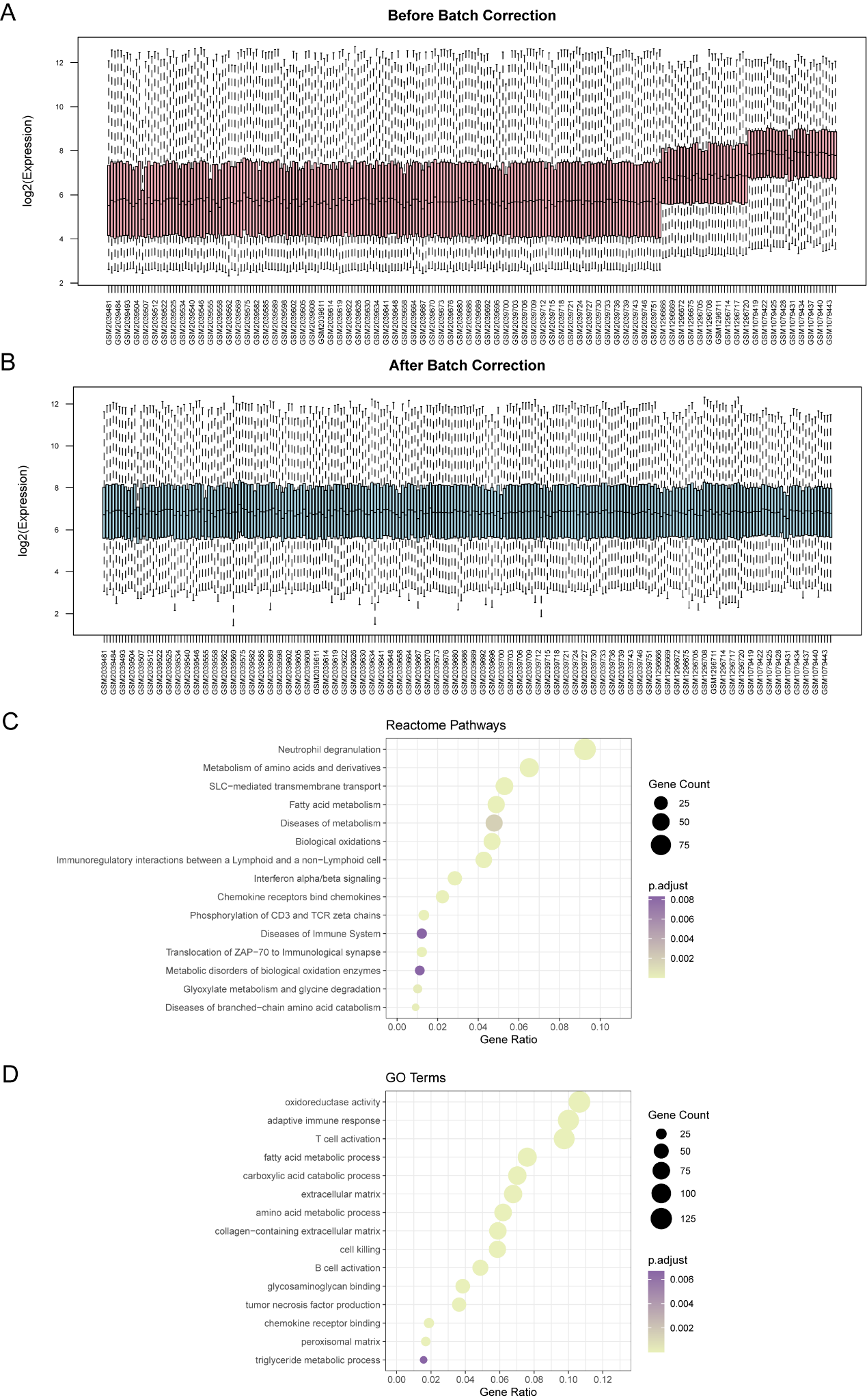
 **Fig. S1.** (A, B) Boxplots showing gene expression distributions before and after batch correction. (C) Reactome enrichment of DEGs. (D) GO enrichment of DEGs.


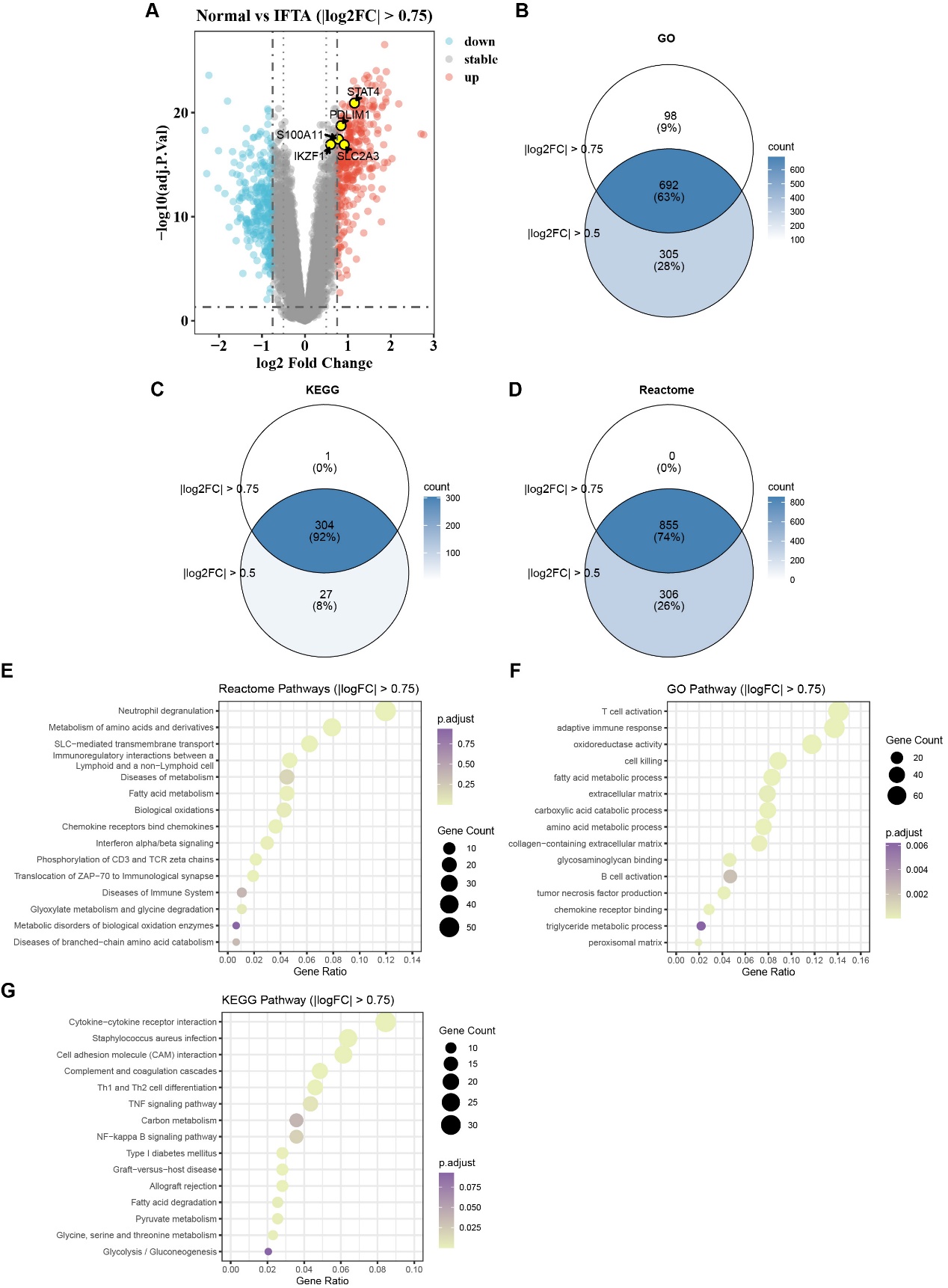


**Fig. S2. Effect of differential-expression thresholds on hub LRGs and pathway enrichment in IFTA.**
(A) Volcano plot of gene expression differences between Normal and IFTA allograft biopsies using a |log₂FC| > 0.75, adj.p<0.05 cutoff. Red and blue dots indicate significantly up- and down-regulated genes, respectively; grey points denote non-significant changes. The five hub lactylation-related genes (STAT4, PDLIM1, S100A11, IKZF1 and SLC2A3) are highlighted. Outer thick dashed vertical lines=0.75; inner thin dashed vertical lines=0.5 (B–D) Venn diagrams comparing pathways identified at two differential-expression thresholds (|log₂FC| > 0.75 vs. |log₂FC| > 0.5, adj.p<0.05) for (B) GO terms, (C) KEGG pathways, and (D) Reactome pathways. Numbers and percentages indicate the proportion of pathways unique to each threshold or shared between both, illustrating that most biologically relevant pathways are retained across a range of fold-change cutoffs. (E) Reactome enrichment of DEGs. (F) GO enrichment of DEGs. (G) KEGG enrichment of DEGs


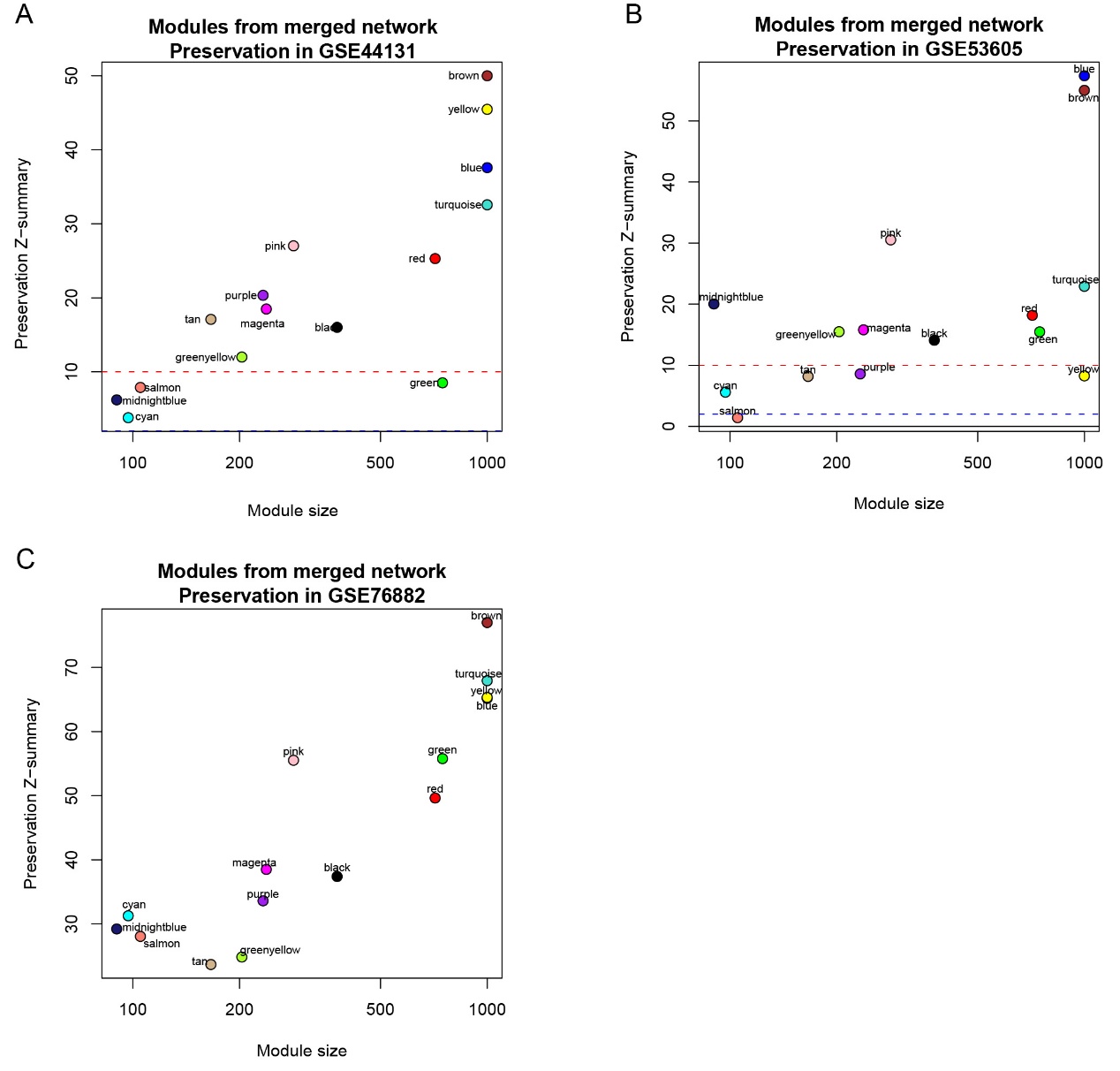


**Fig. S3. Preservation of WGCNA modules across independent renal allograft cohorts.**
**(A–C)** Z-summary statistics of module preservation for gene co-expression modules derived from the merged discovery network, evaluated separately in **(A)** GSE44131, **(B)** GSE53605, and **(C)** GSE76882. Each point represents one module, with color names corresponding to module labels and x-axis indicating module size. The horizontal blue (Z = 2) and red (Z = 10) dashed lines mark thresholds for low, moderate, and strong preservation, showing that most key modules are robustly preserved across independent datasets.


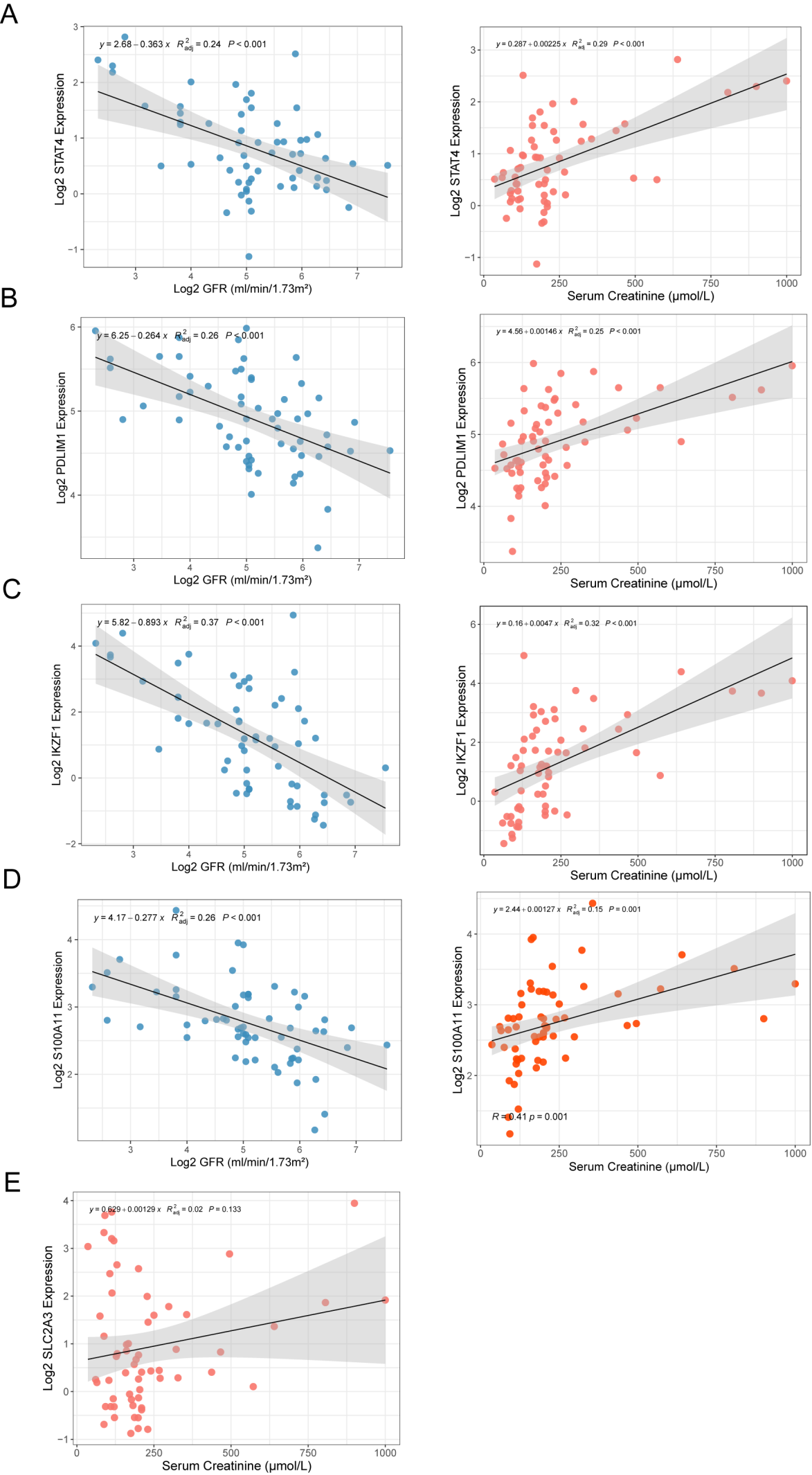


**Fig. S4. Clinical correlation between hub lactylation-related genes and renal function.** (A–E) Correlation between expression of five hub genes (STAT4, SLC2A3, PDLIM1, IKZF1, S100A11) and renal functional parameters. Left: gene expression vs. eGFR. Right: gene expression vs. serum creatinine.


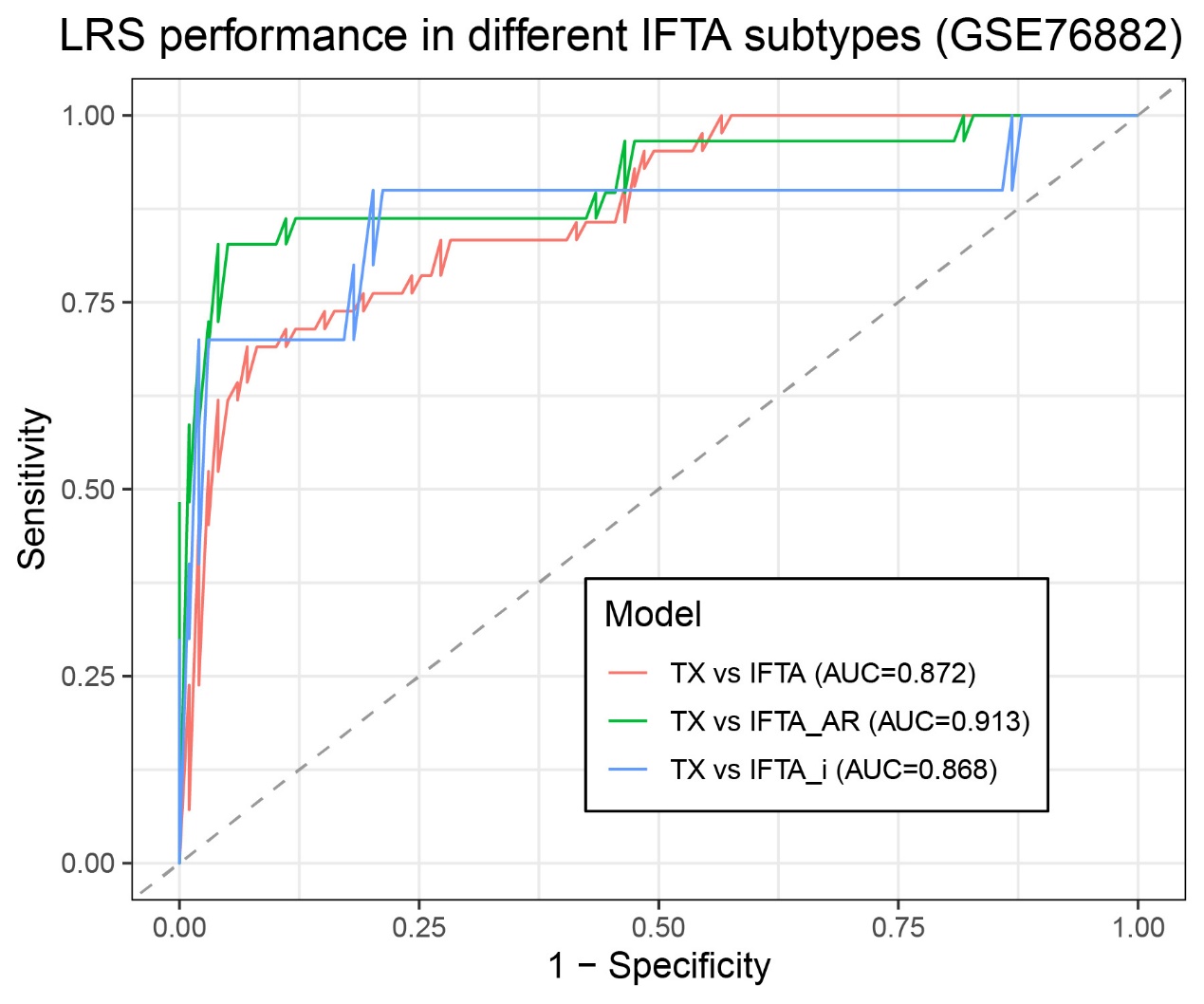


**Fig. S5. Lactylation-related risk score (LRS) performance in different IFTA subtypes (GSE76882).**
Receiver operating characteristic (ROC) curves showing the ability of the LRS to distinguish stable grafts (TX) from classic IFTA (red), IFTA with acute rejection (IFTA_AR, green), and IFTA with interstitial inflammation (IFTA_i, blue) in the GSE76882 cohort. The corresponding AUC values for each comparison are indicated in the legend.


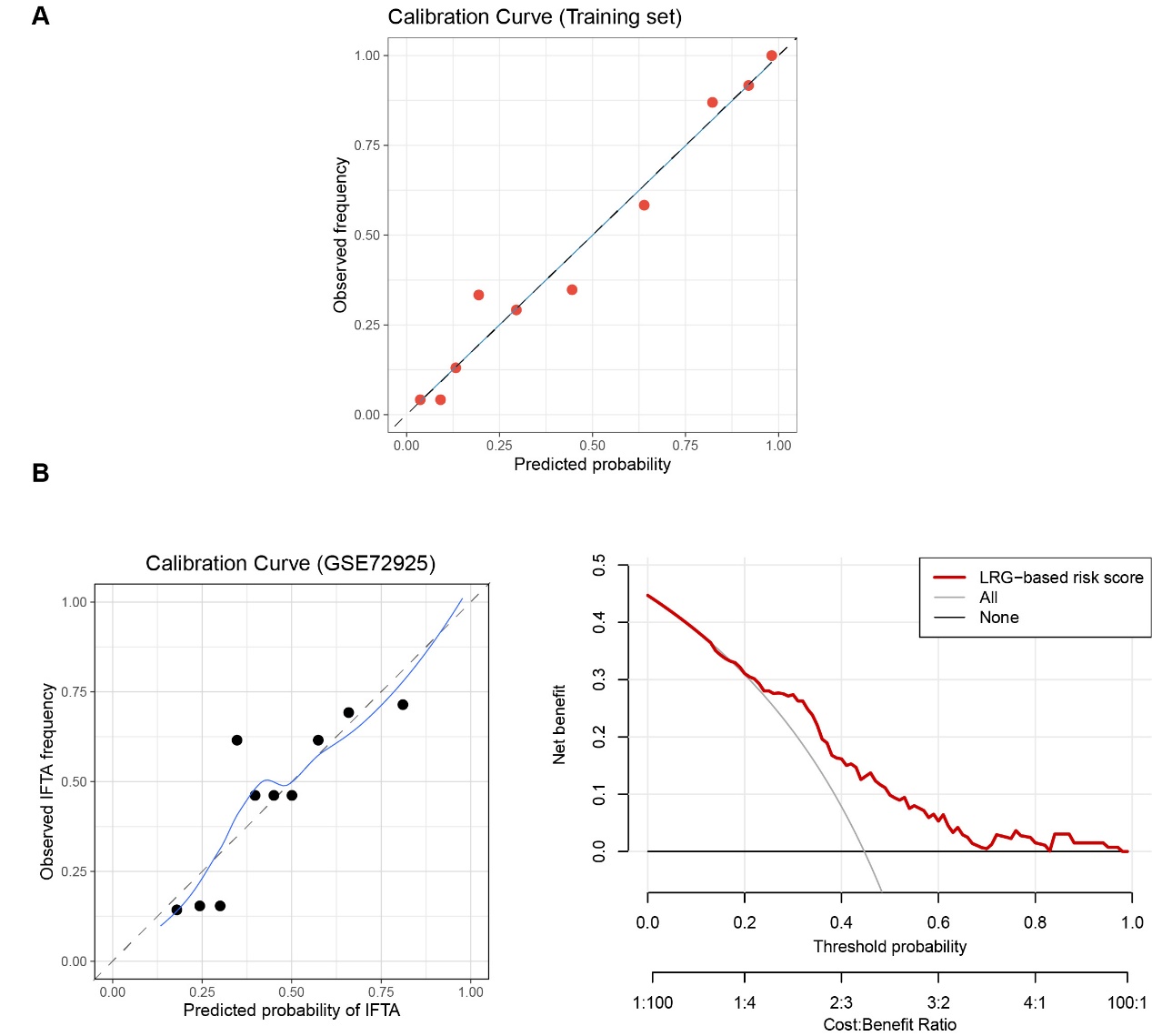


**Fig. S6. Calibration and clinical utility of the LRG-based risk score.**
**(A)** Calibration curve of the lactylation-related risk score (LRS) in the training cohort, showing close agreement between predicted probabilities and observed frequencies of IFTA.
**(B)** Calibration (left) and decision-curve (right) analyses of the LRS in the independent validation cohort GSE72925. The calibration plot indicates generally acceptable agreement between predicted and observed IFTA risk, while the decision-curve analysis demonstrates that the LRS provides greater net clinical benefit than “treat-all” or “treat-none” strategies across a broad range of


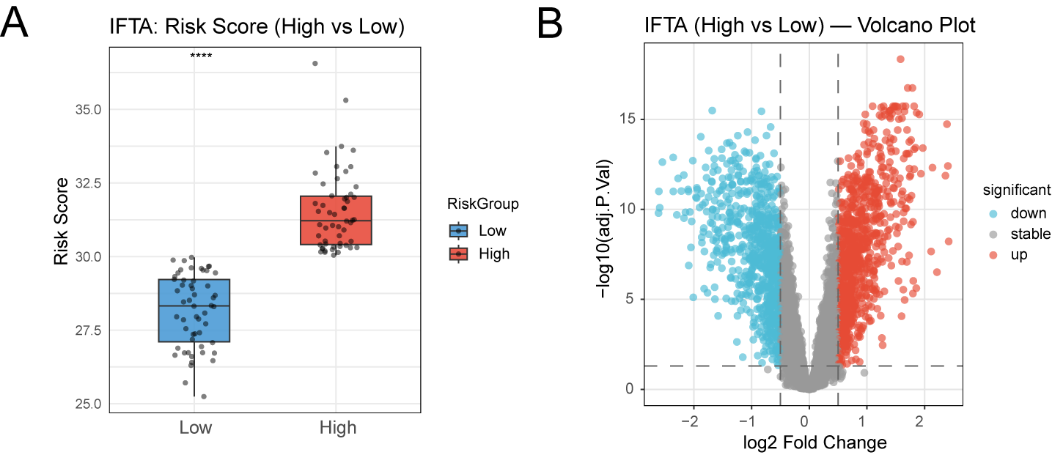


**Fig. S7.** (A) Boxplot showing risk score distribution in IFTA patients divided into high- and low-risk groups. (B) Volcano plot displaying differentially expressed genes (DEGs) between high- and low-risk groups.


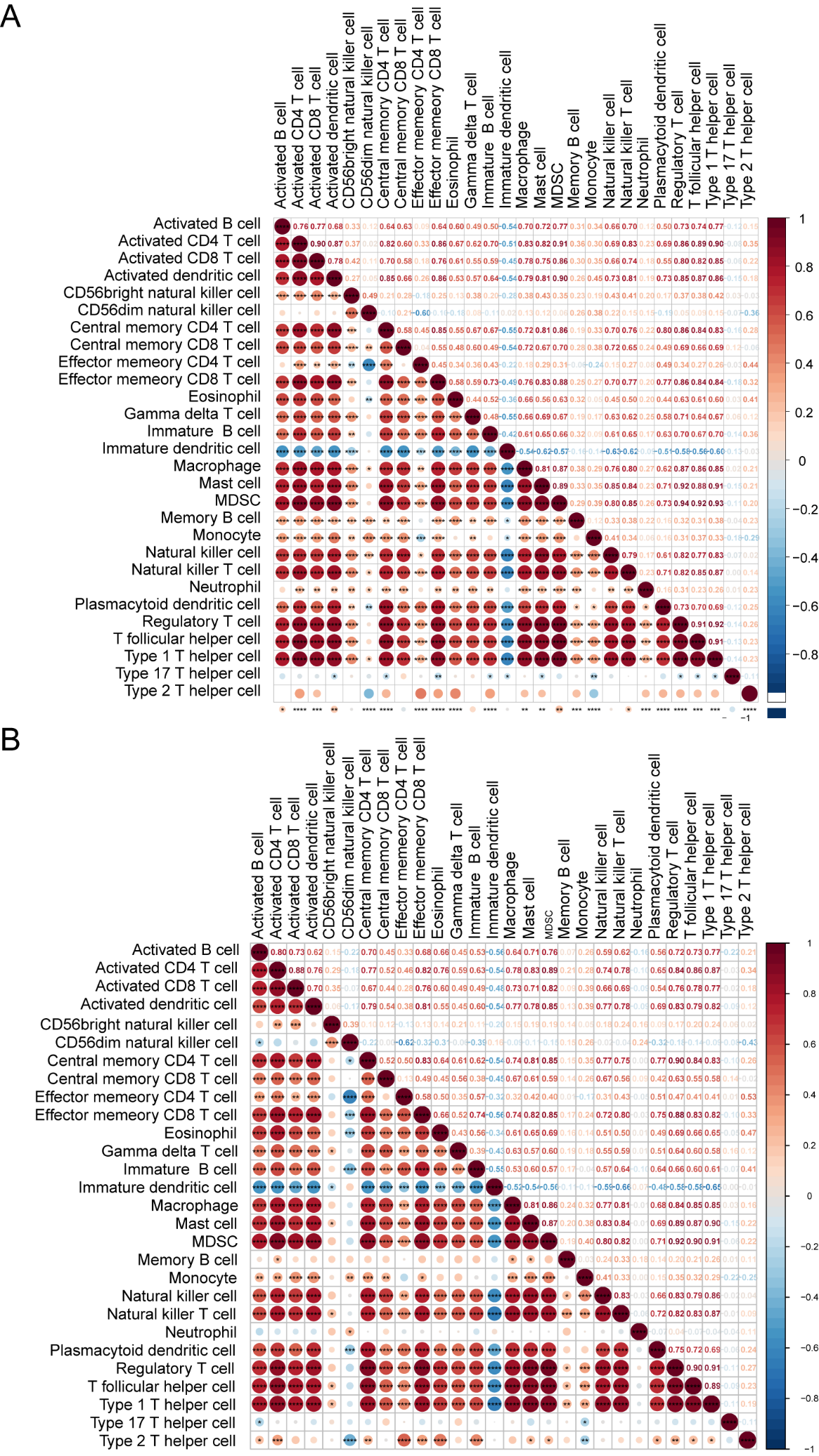


**Fig. S8. Immune–immune correlation maps.** (A) Pairwise correlations among 28 immune cell types in Normal and IFTA cohorts (computed from ssGSEA scores). Dot color encodes Spearman’s *r* (red, positive; blue, negative; scale −1–1) and dot size reflects significance (−log10(FDR)); diagonal indicates self-correlation. (B) Pairwise correlations among the same immune cell types in low-LRS and high-LRS groups, displayed as in (A).


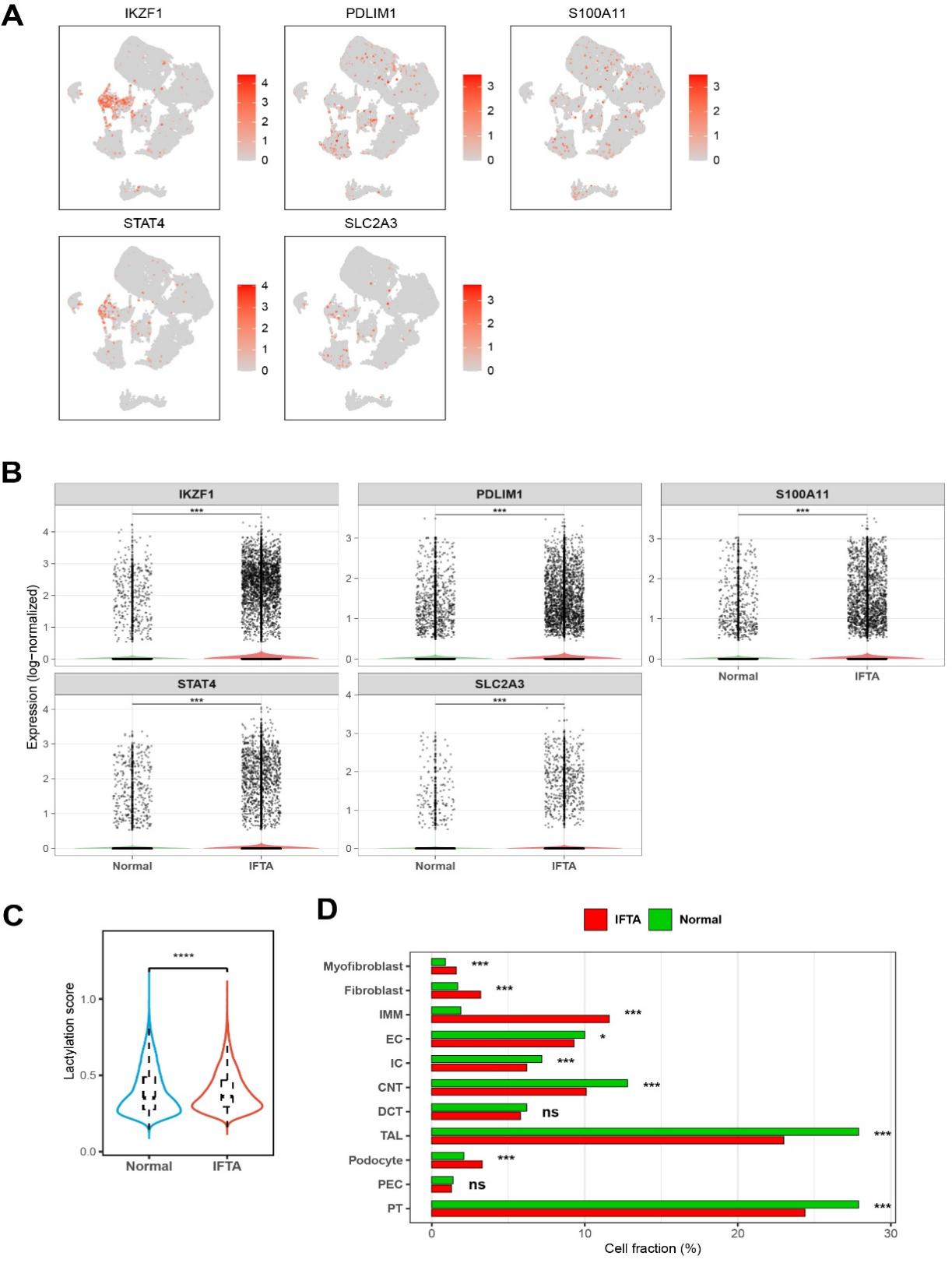


**Figure S9**. **Hub LRG expression and lactylation scores in kidney allograft snRNA-seq**. (A) UMAP feature plots showing per-nucleus expression of the five hub LRGs (*IKZF1*, *PDLIM1*, *S100A11*, *STAT4*, *SLC2A3*); color indicates scaled expression. (B) Dot/box plots comparing log-normalized expression of each hub gene between Normal and IFTA nuclei. Lines denote median and IQR; significance by two-sided Wilcoxon test. (C) Violin plot of the lactylation score , higher in IFTA than Normal. *p < 0.05, **p < 0.01, ***p < 0.001; ****p < 0.001; ns: not significant. (D) Bar plots showing the relative abundance of each annotated cell type in Normal versus IFTA biopsies; bars represent mean cell fraction per group (red, IFTA; green, Normal).*p < 0.05, **p < 0.01, ***p < 0.001, ****p < 0.0001; ns, not significant.


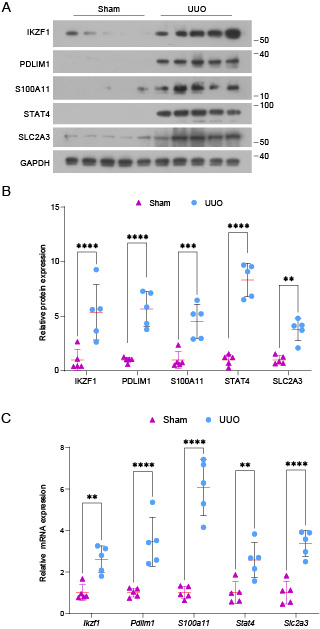


**Figure S10 Experimental validation of lactylation-related hub genes in a murine UUO model.** (A) Representative Western blots showing protein levels of IKZF1, PDLIM1, S100A11, STAT4, and SLC2A3 in Sham and UUO kidneys, with GAPDH as the loading control. (B) Densitometric quantification of Western blot signals normalized to GAPDH. (C) qPCR analysis demonstrating increased mRNA expression of Ikzf1, Pdlim1, S100a11, Stat4, and Slc2a3 in UUO kidneys compared with sham controls. For each group, 5–6 mice were analyzed; the average value per mouse was used as one biological replicate for statistical analyses. Data are mean ± SD; *p < 0.05, **p < 0.01, ***p < 0.001, **p < 0.0001; ns, not significant.
