# Supplementary material for: Identification of lactylation and its hub genes in contributing immune activation and renal allograft fibrosis by integrative bioinformatics and machine learning
Source: Front Immunol. 2026 Feb 10;17:1741864. doi: 10.3389/fimmu.2026.1741864 (PMC12932934; doi:10.3389/fimmu.2026.1741864)
Supplement: Supplementary file 5 [file Table2.docx]

Table S1

Summary of the data sets utilized in this research and their features.

| **Dataset** | **Database** | **Platform** | **Normal vs IFTA** | **Tissue** |
| --- | --- | --- | --- | --- |
| [GSE44131](https://www.ncbi.nlm.nih.gov/geo/query/acc.cgi?acc=GSE44131) | GEO | [GPL6244](https://www.ncbi.nlm.nih.gov/geo/query/acc.cgi?acc=GPL6244) | 12 vs 17 | Kidney allograft biopsies |
| [GSE53605](https://www.ncbi.nlm.nih.gov/geo/query/acc.cgi?acc=GSE53605) | GEO | [GPL571](https://www.ncbi.nlm.nih.gov/geo/query/acc.cgi?acc=GPL571) | 18 vs 10 | Kidney allograft biopsies |
| [GSE76882](https://www.ncbi.nlm.nih.gov/geo/query/acc.cgi?acc=GSE76882) | GEO | [GPL13158](https://www.ncbi.nlm.nih.gov/geo/query/acc.cgi?acc=GPL13158) | 99 vs 81 | Kidney allograft biopsies |
| [GSE72925](https://www.ncbi.nlm.nih.gov/geo/query/acc.cgi?acc=GSE72925) | GEO | [GPL570](https://www.ncbi.nlm.nih.gov/geo/query/acc.cgi?acc=GPL570) | 73 vs 59 | Kidney allograft biopsies |
| [GSE195718](https://www.ncbi.nlm.nih.gov/geo/query/acc.cgi?acc=GSE195718) | GEO | [GPL24676](https://www.ncbi.nlm.nih.gov/geo/query/acc.cgi?acc=GPL24676) | 3 vs 6 | Kidney allograft biopsies |
