# Supplementary material for: Identification of lactylation and its hub genes in contributing immune activation and renal allograft fibrosis by integrative bioinformatics and machine learning
Source: Front Immunol. 2026 Feb 10;17:1741864. doi: 10.3389/fimmu.2026.1741864 (PMC12932934; doi:10.3389/fimmu.2026.1741864)
Supplement: Supplementary file 7 [file Table4.docx]

**Supplemental Table 3. List of primers used in this study**

| *Stat4*-F (mouse) | CCTGCGAGACTACAAGGTTATC |
| --- | --- |
| *Stat4* -R (mouse) | CTTTGGGAATGTCAGGGTAGAG |
| *Pdlim1*-F (mouse) | ACAACCTCTCGCCATTTCC |
| *Pdlim1* -R (mouse) | GTGTCATGCTGCTGGTATCT |
| *S100a11*-F (mouse) | CCTTGACCGCATGATGAAGA |
| *S100a11*-R (mouse) | GCTATAGCTAAGCCACCAATGA |
| *Ikzf1*-F (mouse) | TTGTGGCCGGAGCTATAAAC |
| *Ikzf1* -R (mouse) | TGCCATCTCGTTGTGGTTAG |
| *Slc2a3*-F (mouse) | AGGAGGAAGACCAAGCTACA |
| *Slc2a3* -R (mouse) | GTGACATCCGAACACTCTCATC |
| *Gapdh*-F (mouse) | GCAAATTCAACGGCACAGTCAAG |
| *Gapdh*-R (mouse) | TCGCTCCTGGAAGATGGTGATG |
